# *In vivo* studies of a peptidomimetic that targets EGFR dimerization in NSCLC

**DOI:** 10.7150/jca.46320

**Published:** 2020-08-18

**Authors:** Leeza Shrestha, Sitanshu S. Singh, Pravin Parajuli, Achyut Dahal, George Mattheolabakis, Sharon Meyer, Joydeep Bhattacharjee, Seetharama D. Jois

**Affiliations:** 1 School of Basic Pharmaceutical and Toxicological Sciences, College of Pharmacy, University of Louisiana Monroe, Monroe, LA 71201.; 2 Biology Program, School of Sciences, University of Louisiana, Monroe, Monroe, LA 71029.

**Keywords:** NSCLC, HER2, EGFR, dimerization, peptidomimetic

## Abstract

Studies related to lung cancer have shown a link between human epidermal growth factor receptor-2 (HER2) expression and poor prognosis in patients with non-small cell lung cancer (NSCLC). HER2 overexpression has been observed in 3-38% of NSCLC, while strong HER2 protein overexpression is found in 2.5% of NSCLC. However, HER2 dimerization is important in lung cancer, including EGFR mutated NSCLC. Since HER2 dimerization leads to cell proliferation, targeting the dimerization of HER2 will have a significant impact on cancer therapies. A peptidomimetic has been designed that can be used as a therapeutic agent for a subset of NSCLC patients overexpressing HER2 or possessing HER2 as well as EGFR mutation. A cyclic peptidomimetic (**18**) has been designed to inhibit protein-protein interactions of HER2 with its dimerization partners EGFR and HER3. Compound **18** exhibited antiproliferative activity in HER2-positive NSCLC cell lines at nanomolar concentrations. Western blot analysis showed that **18** inhibited phosphorylation of HER2 and Akt *in vitro* and *in vivo*. Stability studies of **18** at various temperature and pH (pH 1 and pH 7.6), and in the presence of liver microsomes indicated that **18** was stable against thermal and chemical degradation. Pharmacokinetic parameters were evaluated in nude mice by administrating single doses of 4 mg/kg and 6 mg/kg of **18** via IV. The anticancer activity of **18** was evaluated using an experimental metastasis lung cancer model in mice. Compound **18** suppressed the tumor growth in mice when compared to control. A proximity ligation assay further proved that **18** inhibits HER2:HER3 and EGFR: HER2 dimerization. Overall, these results suggest that **18** can be a potential treatment for HER2-dimerization related NSCLC.

## Introduction

Lung cancer is the second most common cancer in men after prostate cancer and in women after breast cancer. Lung cancers are of two types; non-small cell lung cancer (NSCLC), which constitutes 80-85% of lung cancers, and small cell lung cancer (SCLC), which constitutes 10-15% of lung cancers 1. Although cancer therapies using tyrosine kinase inhibitors (TKIs), immunotherapy, and combination therapy have led to improved survival benefits in selected NSCLC patients, the overall survival rate is still low 2. When multiplexed assays were performed on the tumors from patients with lung adenocarcinomas to test for oncogenic drivers, an oncogenic driver was found in 64% of the patients 3. Therefore, over the past few decades, NSCLC treatment has evolved from the use of cytotoxic drugs to the use of personalized regimens targeting particular molecular targets such as anaplastic lymphoma kinase (ALK), epidermal growth factor receptor (EGFR), ROS1, vascular endothelial growth factor receptor (VEGFR), human epidermal growth factor receptor-2 (HER2), and Kirsten ras sarcoma (KRAS) 4. Check point blockers such as programmed cell death protein 1-programmed death-ligand 1 (PD1-PDL1) have shown promise for managing NSCLC 5, 6. Monoclonal antibodies and TKIs such as cetuximab, bevacizumab, nivolumab, and pembrolizumab, and crizotinib, erlotinib, afatinib, and gefitinib are approved by the FDA for the treatment of NSCLC 4.

One of the important molecular targets for NSCLC treatment is the EGFR family of receptors. This family is comprised of four members, namely, ErbB1/HER1, ErbB2/HER2, ErbB3/HER3, and ErbB4/HER4. These cell surface proteins play an important role in cell proliferation, differentiation, survival, and migration. Overexpression of HER2 is associated primarily with aggressive types of cancer with poor prognosis and lower survival rates. Reports related to HER2 overexpression in NSCLC vary depending on the detection method used for HER2 quantification 7. HER2 overexpression has been observed in 3-38% of NSCLC, while strong HER2 protein overexpression is found in 2.5% of NSCLC 8, 9. In addition, HER2 mutations have been detected in 2-4% of lung adenocarcinomas 10. While treatment by targeting HER2 over-expression, gene amplification, or mutations remains controversial, there are certain subgroups of patients that have shown good response to HER2 targeted therapy 11-14. Most of the HER2 targeted therapy for NSCLC are in a clinical trial 15. Targeted therapies using TKIs develop resistance within 2-5 years 16. Thus, there is a need to develop novel new ways of treating NSCLC. HER2 dimerization with other members of EGFR has been shown to be important in EGFR mutated NSCLC 17. Thus, we are interested in targeting HER2 dimerization with other EGFR family members such as EGFR and HER3. At present, HER1, HER2, and HER3 are validated targets for the treatment of cancer, and HER4 is mostly associated with cardiac development 18, 19. Previously we have reported the design of a peptidomimetic that targets HER2 receptor domain IV and inhibits HER2 dimerization with other EGFRs 20. Peptidomimetics have some advantages over antibodies as they are more stable, have relatively lower production costs, and are less immunogenic compared to antibodies, and some peptides are even known to be orally available 21, 22.

**Figure 1:**
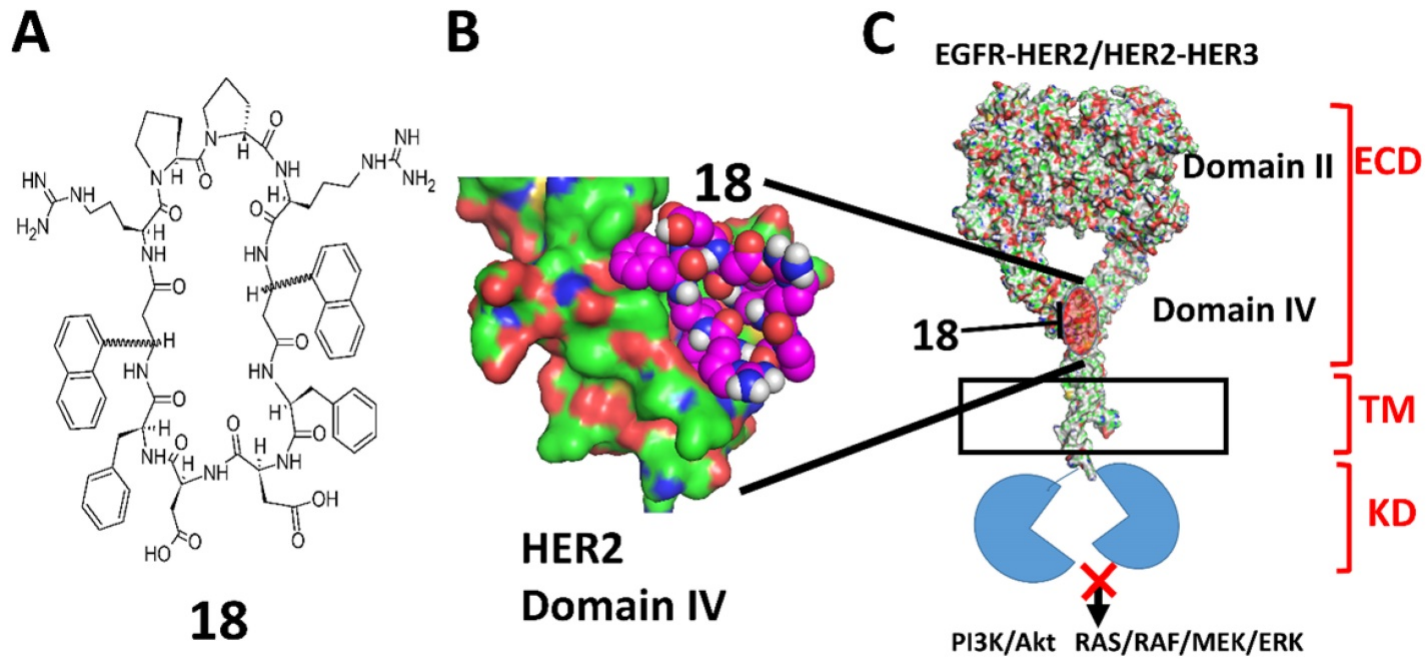
** A)** Chemical structure of 18. 3-amino-3-(1-napthyl propionic acid) (Anapa) can have ***R*** or ***S*** configuration. Active compound has ***R*** configuration at Anapa. **B)** proposed binding site of **18** on C-terminal HER2 domain IV. **C)** Binding of 18 to HER2 and inhibition of EGFR-HER2 or HER2-HER3 dimerization and modulation of downstream signaling for cell proliferation by different pathways.

Monoclonal antibodies such as trastuzumab and pertuzumab are effective in HER2 positive breast cancer 23, 24. However, the efficacy of pertuzumab as monotherapy for NSCLC is not clear at present 25, 26. Pertuzumab and trastuzumab, in combination 27 or pertuzumab along with TKIs, seem to have some efficacy in NSCLC 26, 28. Trastuzumab binds to HER2 domain IV and mediates antibody-dependent cell-mediated cytotoxicity, inhibits HER2 mediated cell signaling, HER2 cleavage, angiogenesis, and DNA damage repair whereas, pertuzumab binds to domain II of HER2 and inhibits receptor dimerization, phosphorylation, and activation of downstream cell signaling 29. Although pertuzumab and trastuzumab bind to the HER2 ECD, the mechanisms of action of these two antibodies are different compared to that of the peptidomimetics we designed. The biological activity of **18** (**Fig. 1A**) *in vitro* and *in vivo*, including its anticancer activity, has been evaluated in a breast cancer mouse model and reported previously 30. In this paper, we describe the physicochemical properties of a peptidomimetic (**18**) and evaluate the pharmacokinetic properties as well as the anti-tumor activity in an experimental metastasis lung cancer mouse model. Our studies indicated that **18** exhibited antiproliferative activity in NSCLC cell lines at nanomolar concentrations and inhibited phosphorylation of HER2 as well as downstream signaling proteins such as Akt. Pharmacokinetic studies indicated that **18** exhibits good stability *in vivo* with a terminal half-life of more than 40 h. *In vivo* studies in a mouse model of NSCLC indicated that **18** delayed tumor growth progression. The overall study suggests that **18** can be a potential therapy for targeting HER2 overexpressed NSCLC.

## Materials and Methods

### Cell lines

HER2 positive A549 lung cancer cell line was purchased from American Type Culture Collection (ATCC, Manassas, VA) and subcultured in RPMI media (ATCC) with 10% FBS. A549-Red-FLuc Bioware® Brite Cell Line was purchased from PerkinElmer and subcultured in RPMI-1640 media (ATCC) with 10% FBS. Cells were incubated in a humidified atmosphere of 5% CO_2_ at 37°C.

### Antiproliferative activity

Antiproliferative activity of **18** on A549 cells was measured using CellTiter-Glo® assay, as described in our previous publications. In a 96-well plate, 1 × 10^4^ cells/well were seeded and incubated overnight at 37 °C and 5% CO_2_. The stock solution of the peptidemimetic was prepared by dissolving them in DMSO. Stock solutions of the compound and controls were diluted using the serum-free medium to prepare solutions of different concentrations of compounds with DMSO concentration not exceeding 1% (v/v) in the final solution in each well of a 96-well plate. Compound **18** in the medium was added to the wells in triplicate and incubated for 72 h, and luminescence was read using CellTiter-Glo assay 31. A dose-response curve was generated using percentage cell viability vs. log concentration of compounds, and IC_50_ values were obtained using Prism® (GraphPad Software). Experiments were repeated three times to obtain standard deviation values.

### Western blot

A549 cells were treated with 2 µM **18** or 2 µM lapatinib (positive control). Cells without treatment were employed as a negative control. Cells were treated for 36 h, washed, and trypsinized. 35 μg of protein from each sample was loaded on Novex® 4-20% tris-glycine gels and transferred into nitrocellulose membranes. The membranes were blocked with 2% bovine serum albumin solution and incubated with primary antibodies overnight at 4 °C. Antibodies for the detection of total HER2 protein (t-HER2), phosphorylated HER2 protein (p-HER2), total AKT (t-AKT); (Cell Signaling Technology, phosphorylated AKT (P-Akt, S473); (Cell Signaling Technology) were used at 1:1000 dilution. Secondary antibodies with HRP conjugation was incubated for 1 h. After the addition of the substrate and enhancer solutions from a super signal enhanced chemiluminescence kit (Pierce, Rockford, IL) to the membrane, images were captured using ChemiDoc™ Touch Gel Imaging System (Bio-Rad), and band densities were quantified. A representative Western blot image was used for the final presentation. Data are from triplicates and are presented as the mean ± standard error of the mean (SEM). To evaluate the significance, statistical analysis was performed by a one-tail T-test using Prism Graph Pad (GraphPad Software, San Diego). *P<* 0.05 was considered statistically significant 20, 32.

### Circular dichroism spectroscopy

Circular dichroism experiments were done using a Jasco J-815 CD Spectrometer (Jasco, Japan) at temperatures from 20 to 80 °C with a gradual 5 °C increase before reading. The sample was prepared by dissolving **18** in a few microliters of methanol and then adding water to obtain a concentration of 18 μM. The spectra for samples were recorded from 185-350 nm wavelengths with a total of four scans for each temperature, and wavelength vs. ellipticity was plotted for an average of four scans.

### HPLC analysis

Shimadzu FCV-20AH2 manual injector was used with Ultra C18, 5 µm (250 × 4.6 mm) column, Shimadzu SPD-20A UV/Vis detector, and Shimadzu LC-20AP pump. 40% acetonitrile (0.1% TFA) with 60% water (0.1% TFA) was used as mobile phase. Relative Peak area was plotted as % remaining compound with respect to time compared with zero time point, and data are from triplicate experiments. For pH stability, a buffer with pH 1 was obtained by adding 0.1 M HCl, and a buffer with pH 7.5 was obtained by adding 0.1 M PBS (Sigma).

### Liver microsomal stability assay

A microsomal metabolic stability assay was conducted using human mixed pooled microsome (Corning® UltraPool™ HLM 150). The reaction mixture consisted of human liver microsome (0.5 mg/mL), **18** (10 µM), and cofactor solution (10 mM NADPH in 0.1 M PBS). Incubations were done at 37 °C for 6 h. Samples of 250 µL volume were withdrawn from the reaction mixture at 0, 0.25, 0.5, 1, 2, 3, 4, and 6 h and transferred into new tubes. The reaction was stopped by protein precipitation through the addition of 1 mL of acetonitrile. The sample was then analyzed using HPLC and mass spectrometry.

### Stability in simulated gastric and intestinal fluids

Simulated intestinal fluid TS and simulated gastric fluid were purchased from Ricca Chemical (Arlington, TX). Pepsin (0.32 g/100 ml) was added to the simulated gastric fluid. To determine the effect of simulated gastric and intestinal fluids on **18**, it (10µM) was incubated in gastric fluid with pepsin for 6 h and intestinal fluid for 12 h at 37 °C. Samples were taken at particular time intervals and analyzed using the HPLC method 33. Methods used were similar to the methods explained above for HPLC analysis.

### Mass spectrometry

A sample solution was prepared by adding 100 μL methanol into a freeze-dried sample. A saturated solution of α-cyano-4-hydroxycinnamic acid (CHCA; Sigma-Aldrich, St. Louis, MO, USA) dissolved in a mixture of 50/50 (v/v) of acetonitrile and 0.1% trifluoroacetic acid (TFA) in water was used as the matrix. MALDI-TOF MS measurements were performed using a high-resolution mass spectrometry instrument (Ultraflextreme, Bruker Daltonics, Billerica MA).

### Surface plasmon resonance analysis

Surface plasmon resonance analysis was performed in 0.01 M phosphate-buffered saline (NaCl 0.138 M; KCl -0.0027 M); Tween® 20 -0.05%, pH 7.4 using a Reichert Life Sciences System. Immobilization of HSA to the hydrophilic carboxymethylated dextran matrix of the CM5 sensor chip was carried out by the standard primary amine coupling method. Different concentrations of **18** were used as analyte. All binding experiments were performed at a flow rate of 25 µL/min. After each interaction analysis, the sensor chip was regenerated with water. A blank subtracted sensorgram for **18** was represented. The K_d_ values were obtained using the 1:1 kinetic evaluation fitting tool of Tracedrawer software.

### Molecular docking

The crystal structure of human serum albumin (HSA) was obtained from the RCSB Protein Data Bank (PDB code: 2BXD). The structure of **18** was obtained from our previous work 20. The binding of **18** to and HSA (PDB ID:2BXD) was modeled using AutoDock 4.0, and Lamarckian genetic algorithm (LGA) in Autodock was used to estimate the possible binding conformations of **18** with HSA^34^. While docking, 10 different conformations were considered for **18** bindings to HSA. The conformer with the lowest binding free energy was used for further analysis. Final structures were converted into PDB files and visualized using PyMOL Molecular Graphics System (Schrodinger Inc. OR).

### *In vivo* studies

Female Foxn1-nude, 6-7 week-old mice (Envigo) were used in all studies. All animals were handled according to the approved protocol from IACUC at the University of Louisiana at Monroe.

### Pharmacokinetics study

Pharmacokinetic parameters were evaluated in nude mice by administrating single doses of 4 mg/kg and 6 mg/kg of **18** via IV. After administration of the compound, mice were sacrificed at certain time intervals (0, 0.25, 0.75, 2, 4, 6, 12 and 24 h), and blood samples were taken to study the amount of compound remaining in blood circulation. The samples were then centrifuged at 1000 rpm for 5 min. The separated supernatant serum samples were precipitated by acetonitrile to extract the analytes and passed through the Sep-Pak (Waters) column. Chromatographic separation was performed on an HPLC. Mass spectrometry was used to identify the correct molecular ion of the intact compound.

### Antitumor assessment

All animal studies were conducted in accordance with NIH guidelines for care, and use of laboratory animals and protocols were approved by the Institutional Animal Care and Use Committee at the University of Louisiana Monroe. Bioware® Brite Cell Line A549 Red-FLuc cells that had been engineered to express firefly luciferase (Perkin Elmer) were injected into the tail vein (4.5 × 10^6^ cells in 200 µl PBS per mouse) of 6-week-old Foxn1^nu^*-*nude mice. For bioluminescence imaging, mice were anesthetized with 1-3% isoflurane and 150 mg/kg of D-luciferin (PerkinElmer) in phosphate-buffered saline (PBS) was given by intraperitoneal injection. 8 min after injection, bioluminescence was imaged with an IVIS Lumina III Series (PerkinElmer), and regions of interest (ROI) were drawn around the bioluminescent signals and quantified as photons/second (p/s). Mice with tumors were divided into three groups viz. control (vehicle), 18 treatment, and lapatinib treatment. After two weeks of the inoculation of tumor cells, mice from the respective groups were injected intravenously with **18** (6 mg/kg) or lapatinib (10 mg/kg) or vehicle twice a week for the next three weeks. To evaluate the effect of the treatments, tumors were imaged, and bioluminescence was measured once a week. On day 35, the mice were sacrificed, and lungs, kidney, liver, and heart were removed washed and fixed with 10% buffered formalin and stored. The sections of lung tumor tissue used for Western blot were rinsed with cold PBS and stored at -80 °C.

### Proximity ligation assay (PLA)

The lung tissue samples with tumors were fixed on the slides. PLA assay was done with the lung tissue sample in order to evaluate the inhibition of EGFR:HER2 and HER2:HER3 dimerization. Antigen retrieval on the tissue section of slides was done in a steaming sodium citrate buffer (10 mM, 0.05% Tween 20, pH 6.0) for 15 min. Tissue slides were incubated with primary antibodies overnight at 4 °C and washed, and secondary antibodies PLA+ and PLA- were added. After incubation and washing, PLA detection reagents were added 35, 36. The mounting medium was then added, and the slides were coverslipped. Slides were viewed using an Olympus BX63 fluorescence microscope fitted with deconvolution optics using DAPI, and Texas Red filters. Images were obtained at 40×.

### Histopathology

For histopathological analysis, lungs, kidney, liver, heart, and pancreas were excised from the mice and fixed with 10% normal formalin solution. These fixed samples were embedded in paraffin, and slides were prepared by cutting them into 5 μm slices and mounting on the glass. Prepared slides were stained using hematoxylin and eosin (H&E) staining. Slides were visualized under a microscope (Nikon labophot 2. Type120) and pictures were taken at different magnifications for analysis. Representative pictures from histopathology analysis were used for presentation.

## Results

### Design of the peptidomimetic compound 18

Previously in our lab, peptidomimetics were designed that could bind to domain IV, inhibit the homo or hetero-dimerization, and block HER2-mediated cell signaling 37. The design of the peptidomimetics is based on the spatial disposition of electrostatic and hydrophobic interaction sites in the crystal structure of HER2 protein complexed with its antibody trastuzumab. Using a rational drug design approach, linear peptidomimetics named compounds **5** and **9** were designed and synthesized that exhibited antiproliferative activity in HER2 positive cancer cell lines 37. To prevent enzymatic degradation, compound 9 was cyclized using a repeated sequence and cyclization approach using an L-Pro-D-Pro sequence. In this program, several linear, cyclic as well as conformational constrained peptidomimetics were designed, and *in vitro* activity were evaluated. Among these, compounds **5**, **9,** and **18** were selected for *in vivo* activity based on their IC_50_ value in HER2 positive cancer cell lines. Structure-activity relationships of **18** (**Fig. 1**) and analogs and their effect on HER2-positive breast cancer were described in our previous reports 20, 30, 38-42.

### Compound 18 inhibits cellular proliferation and reduces the phosphorylation of HER2 kinase in A549 cell line

**Figure 2:**
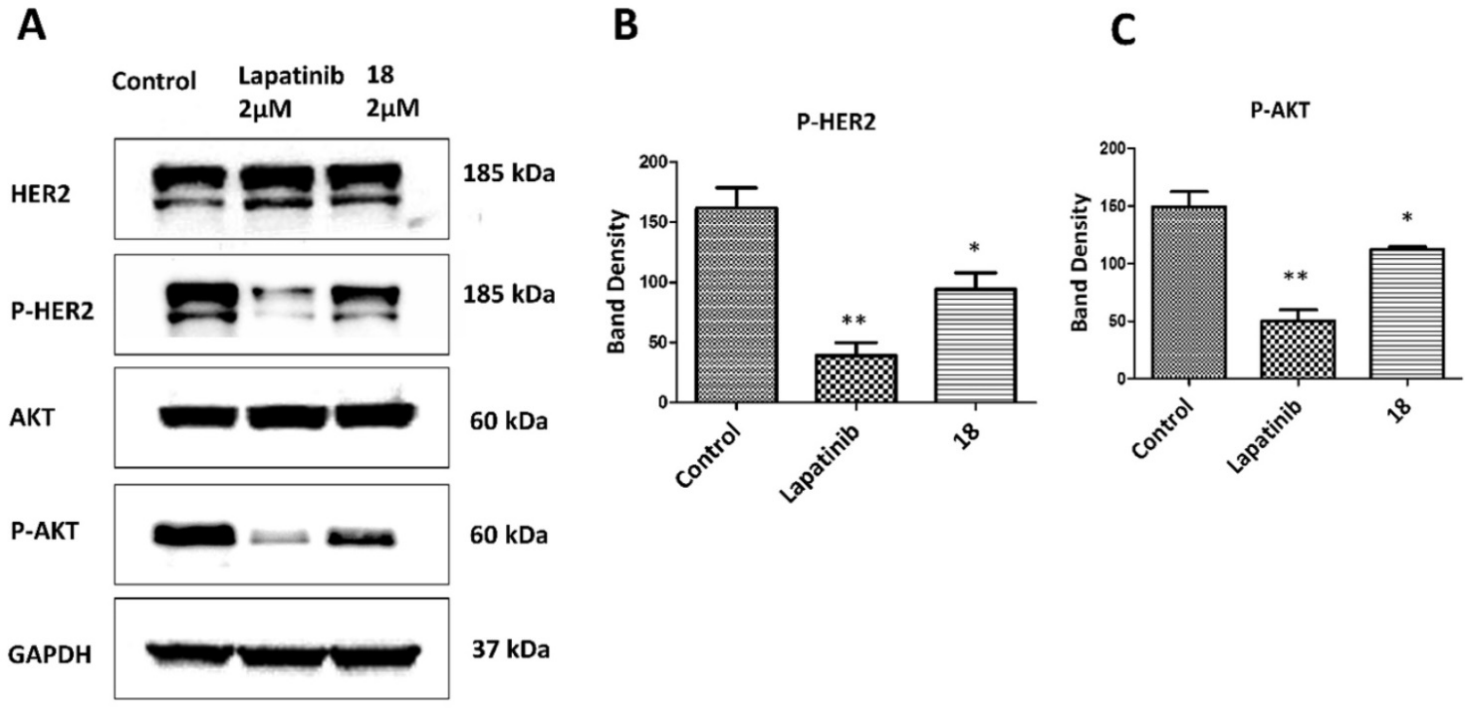
Effect of **18** on phosphorylation of HER2 and Akt. A549 lung cancer cell lines were incubated with **18** and after 36 h, cells were washed and lysed. Western blot was performed to determine p-HER2 and p-Akt as well as total HER2 and Akt. (Blots were cropped and presented). Quantification of signal intensities were done by ImageJ). Lapatinib was used as positive control. **A)** Western blot of HER2, p-HER2, Akt, p-Akt. Equal loading of GAPDH was used for comparison. Experiments were repeated three times. Quantification of **B)** p-HER2 and **C)** p-Akt using densitometry. Band intensity was represented with scaling from GAPDH band intensity. *p<0.05, **p<0.01.

Compound **18** is highly specific for HER2 positive cancer cell lines 20. A549 lung cancer cell lines overexpress HER2 protein and are used as *in vivo* and *in vitro* model of NSCLC. These cells have wild type EGFR and KRAS mutation and are present in about 30% of adenocarcinoma 43-45. Antiproliferative activity of **18** in A549 cell lines was evaluated using CellTiter-Glo assay, and the IC_50_ value was found to be 0.868 ± 0.032 µM (Supporting Information, **Fig. S1**). We have also evaluated the antiproliferative activity of **18** in different cell lines that overexpress HER2 or at basal level and in non-cancerous breast cell lines 20 (**Supporting informationTable S1**). The selectivity of compound **18** is 200 times for HER2 positive cancer cell lines (SKBR-3, BT-474) as compared for non-cancerous cell line MCF-10A. Compared to non-cancerous cells, the antiproliferative activity of compound **18** in HER2 positive lung cancer cell lines Calu-3 is 2000 times and A549 lung cancer cells nearly 50 times selective. For hormonal related breast cancer cells MCF-7, selectivity of compound **18** was more than 60 times and 2000 times less compared to NSCLC cell lines A549 and Calu-3 respectively. To evaluate whether **18** inhibits the phosphorylation of HER2, we carried out Western blots of proteins extracted from A549 cells treated with **18**- and lapatinib. After 36 h of treatment, **18** reduces the phosphorylation by HER2 kinase compared to the control (**Fig. 2ABC**). HER2 phosphorylation is known to induce downstream signaling for cell growth and differentiation (**Fig. 1C**). To study whether **18** inhibits the further down-stream signaling molecules, we evaluated the phosphorylation of Akt. Compound **18** was able to inhibit the phosphorylation of Akt (**Fig. 2C**). Quantitative analysis of the Western blots suggested that **18** significantly inhibited the phosphorylation of HER2 by 41.6% and Akt by 24.7% compared to control. Compared to lapatinib 32, **18** showed less reduction in phosphorylation of HER2 and Akt (**Fig. 2B, C**) as **18** is not a direct inhibitor of tyrosine kinases and inhibition of kinase phosphorylation is due to inhibition of dimerization of EGFR receptors.

### Chemical and thermal stability of compound 18

Peptides can change their conformation upon exposure to high temperatures for a long duration. A rapid increase in temperature to extreme conditions model the accelerated stability test conditions for peptide-like drug substances 46. Circular dichroism spectra of **18** at different temperatures did not show any significant change in the spectra, suggesting that the overall backbone conformation of **18** did not change when the temperature of the solution with **18** increased from 25 to 80 ºC (Supporting Information**Fig. S2**). To confirm the stability of compound **18**, it was incubated at temperatures of 25, 50, and 80 °C for 30 min, and samples were analyzed using reversed-phase HPLC. A plot of peak area corresponding to compound **18** at different temperatures indicated that the there was no significant difference between the peak area calculated at different temperature (**Fig. S3**) and mass spectrometry analysis showed the correct molecular ion (m/z = 1425.70 ± 0.01) for compound **18** at 25, 50 and 80 ºC. These results indicate that compound **18** was intact up to 80 °C (Supporting Information **Fig. S4**).

**Figure 3:**
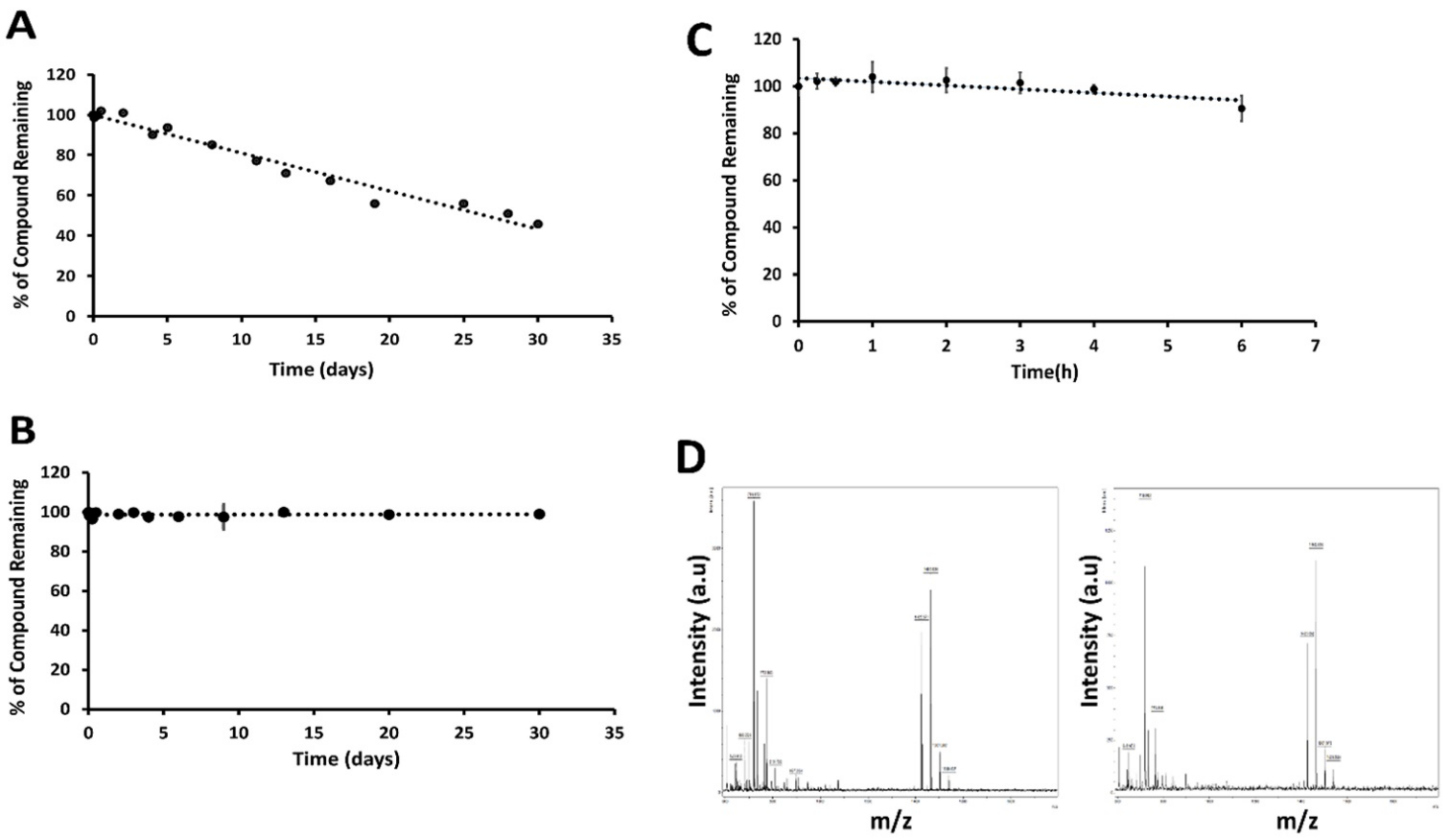
Stability of **18** at different pH. Compound **18** was incubated in 0.1 M HCl (pH 1) and phosphate-buffered saline (PBS, pH 7.5), aliquoted at different intervals, and analyzed by HPLC. Peak area was calculated from HPLC chromatogram. Plot percentage of compound remaining vs. time in days for **A)** pH 1, **B)** pH 7.5. Compound **18** indicated degradation at pH 1 but was stable for 30 days in PBS at pH 7.5. *In vitro* stability of **18** in the presence of human liver microsomes. **C)** Compound **18** was incubated with human liver microsomes, extracted at particular time points and analyzed by HPLC. A plot of relative concentration of **18** with respect to time is shown. For standardization of the assay verapamil was used. **D)** Mass spectra of sample at 0 (left) and 6 h (right) incubated in liver microsomes.

Peptides have limitations for oral administration. If the peptide needs to be administrated via the oral route, the stability of the peptide at different pH conditions is important, as the pH of the digestive system varies from 2 to 7.4 47. To determine the stability of **18** at physiological gastric pH (if given orally) and blood pH (if given intravenously), we incubated **18** at 37 °C in pH 1 and pH 7.5 buffers. Compound **18** was evaluated for its stability for 30 days to have an insight of accelerated stability testing and storage conditions if it has to be formulated in different pH conditions for improving solubility and bioavailability. HPLC analysis of compound **18** in buffers showed that **18** exhibited a decrease of the total intact peptide at pH 1.0 in 30 days (**Fig. 3A**), whereas at pH 7.5 compound was stable for 30 days (**Fig. 3B**). From our studies, it is clear that more than 40% of compound **18** was remaining intact on the 30^th^ day at pH 1.

### Compound 18 is stable in liver microsomes

Human liver microsomes contain a wide variety of drug-metabolizing enzymes and are commonly used to support *in vitro* ADME (Absorption, Distribution, Metabolism, and Excretion) studies and to examine the potential for the first-pass metabolism of orally administered drugs. To evaluate the possibility of oral administration, the stability of **18** was assessed in liver microsomes (Corning, NY) 48. This assay was done by incubating the **18** in the mixture containing liver microsomes in the presence of nicotinamide adenine dinucleotide phosphate (NADPH), the cofactor for cytochrome P450 (CYP) enzymes. Verapamil was used as a positive control. When verapamil was incubated for 2 h, there was a rapid degradation of verapamil, indicating that the liver microsomes were active *in vitro*. When **18** was incubated with liver microsomes for 6 h, it did not show any significant degradation until 6 h (**Fig 3C**). Mass spectrometry analysis of the sample also showed a peak representing **18** at 0 and 6 h time points (**Fig. 3D**). The presence of a significant amount of intact peptide up to 2 hours suggests that the peptide is not rapidly metabolized by the liver by phase I metabolism. This result also suggests the possibility of limited first-pass metabolism for the peptide.

### Compound 18 is stable in simulated gastric fluid and degrades slowly in simulated intestinal fluid

During oral drug delivery, degradation of peptide drugs is possible due to the highly acidic condition of the stomach together with gastric-secreted enzyme pepsin. In addition, enzymes secreted in the intestine, i.e., trypsin, chymotrypsin, elastase, and brush-border membrane-bound enzymes carboxypeptidases A and B can also cause hydrolysis of specific bonds of peptides that leads to peptide degradation and loss of activity. To assess the stability of **18**, it was incubated in gastric fluid with pepsin for 6 h and intestinal fluid for 12 h at 37 °C. The result shows that when incubated with simulated gastric fluid, there was a slight decrease in the concentration of **18;** however, more than 80% of the compound remained in the solution for 6 h (Supporting Information **Fig. S5A)**. Whereas when **18** was incubated with simulated intestinal fluid, there was a significant decrease in the concentration of the compound**,** but more than 40% was still present in the solution at 12 h (Supporting Information**Fig. S5B)**.

### Pharmacokinetic studies

**Figure 4:**
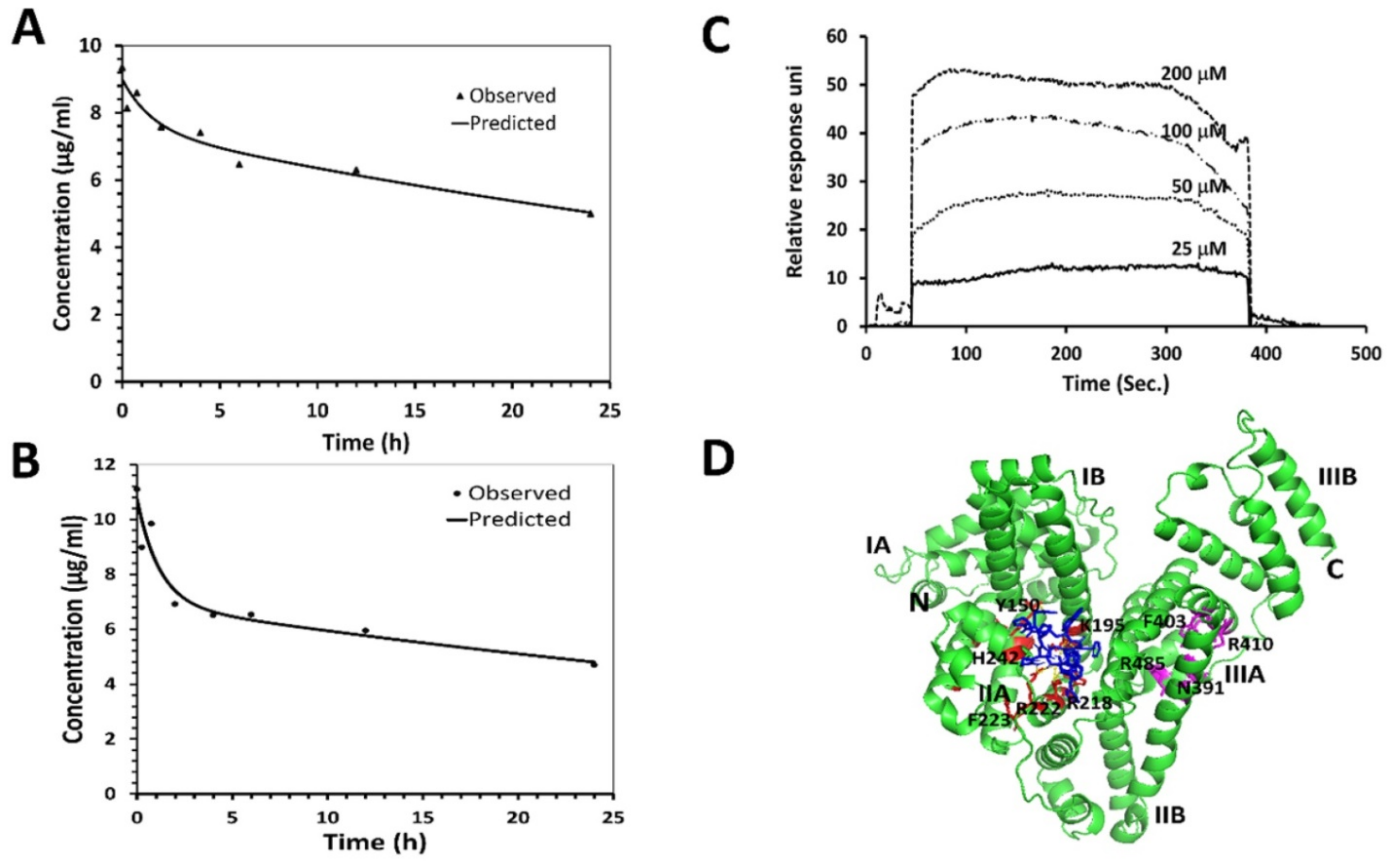
Pharmacokinetic studies of **18** in Foxn1 nude mice. PK studies were performed at two concentrations in mice. Compound **18** in PBS was injected to mice via tail vein, and blood samples were collected at different intervals. Samples were lyophilized after precipitation with cold methanol and analyzed by HPLC. Leuprolide was used as an internal standard. AUC was calculated and concentration of 18 was calculated from a standard curve. Plot of concentration of **18** with time in h **A)** 4 mg/kg and **B)** 6 mg/kg. PKsolver was used to curve fit the data points considering two compartment model. Predicted and observed data points are shown in the graph. Binding of **18** to human serum albumin (HSA) verified by SPR and docking. **C)** HSA was immobilized and **18** was used as analyte. Sensorgram shows the binding of **18** to HSA. **D)** Proposed modes of binding of **18** to HSA using docking. Protein HSA is shown in secondary structure and **18** is shown as blue sticks. Different binding sites of HSA are shown in red and magenta color.

One of the main limitations of peptides *in vivo* is their short half-life in the circulation, which is caused mainly by proteolytic degradation and/or fast renal clearance 49. Cyclic peptides/peptidomimetics are more stable and have a longer half-life than its linear counterparts. The pharmacokinetic profile of **18** was evaluated in nude mice. Compound **18** was administered (single dose) intravenously at 4 mg/kg and 6 mg/kg of body weight to two groups of mice. Blood samples were collected at different time intervals, and analysis of the sample was accomplished using HPLC and mass spectrometry. The concentration-versus-time curves and the results of the pharmacokinetic analysis are summarized in **Figs. 4A, B** (Supporting Information**Fig. S6**). For pharmacokinetic parameter analysis, PKSolver software was used 50. To select an appropriate model with good precision of estimated parameters, PK solver provides several statistical comparison criteria, among which Akaike's information criterion (AIC) and Schwarz criterion (SC) are widely regarded as the most important ones pertinent to our experiments. A model is considered better when AIC and SC are minimum among all the other models 51. Based on the AIC, SC, and curve fitting by the PKSolver software, intravenous bolus administration/two compartmental model was selected for deriving our reported pharmacokinetic parameters. The terminal half-life of **18** following 6 mg/kg and 4mg/kg IV dose administration was predicted to be around 46 and 42 h respectively. Relatively long half-life of **18** in plasma is advantageous since it allows sufficient amount for **18** to reach cancer tissues. Considering that **18** is a peptide/peptidomimetic, and the IC_50_ value in the nanomolar concentration range for HER2 positive lung cancer cells, the half-life is long enough to cause pharmacological action in the body.

### Compound 18 binds weakly with human serum albumin

Human serum albumin (HSA), carrier protein in plasma, plays major roles in pharmacokinetics by binding to most drugs 52, 53. Extensive plasma protein binding limits the amount of compound available to be metabolized and reduces the clearance of the compound. Complete characterization of the mechanism by which drugs bind to proteins such as HSA is important for the pharmacokinetics and pharmacodynamics profiles of drugs. Surface plasmon resonance (SPR)-based binding assay was done to investigate the interaction of **18** with the HSA immobilized on the CM5 sensor chip. Compound **18** at various concentrations (25, 50, 100, and 200 µM) was titrated over the immobilized HSA. As the SPR- sensorgram depicts, the response unit values increased in a concentration-dependent manner, which shows **18** bindings to the immobilized HSA (**Fig. 4C**). The binding kinetic constants were fitted using the 1:1 (Langmuir) binding fitting model, and the equilibrium dissociation constant (K_d_) was determined to be 190 µM indicating weak binding between **18** and HSA. Based on the law of mass action and microscopic reversibility 54, we plotted a graph relating K_d_ of **18** and percentage of drug bound to HSA, assuming albumin concentration in human serum to be 680 µM and **18** concentration to be around 7 µM (equivalent to 10 µg/ml in the pharmacokinetic study). From the relation of the affinity of **18** to the percentage of drug binding to HSA protein, we predicted that 78% of the drug would bind to HSA in serum (Supporting Information **Fig. S7**).

In addition to SPR, computational modeling was done to study the specific binding mode and binding location of **18** with HSA using Autodock 4^34^. The 3D structure of the HSA was obtained from the Protein Data Bank 55. The 3D structure of **18** generated from NMR data and molecular dynamics simulations from our previous publication was used 20. Among the docked structures, the lowest docking energy structure was selected for further analysis. HSA has three domains viz., Domain I, Domain II, and Domain III, and each domain consists of two subdomains, subdomain A and subdomain B (**Fig. 4D**). Subdomains IIA and IIIA have two ligand-binding hydrophobic pockets referred to as site I and site II, respectively 55, 56. The docking result shows that **18** binds to subdomain IIA (site I) of HSA. Binding of **18** to HSA involves hydrogen bonding and hydrophobic interactions, as shown in **Fig. 4D**. The lowest energy docked structure indicated that amino acids K195, R218, R222, E294, R218, A191, and P441 of HSA interact with **18** via hydrogen bonds and hydrophobic interactions. Compound **18** binds near to the warfarin binding site on HSA, as shown by docking studies. The calculated binding free energy for the docked structure was -7.67 Kcal/mol.

### Compounds 18 inhibits tumor growth in experimental metastasis lung tumor model

**Figure 5:**
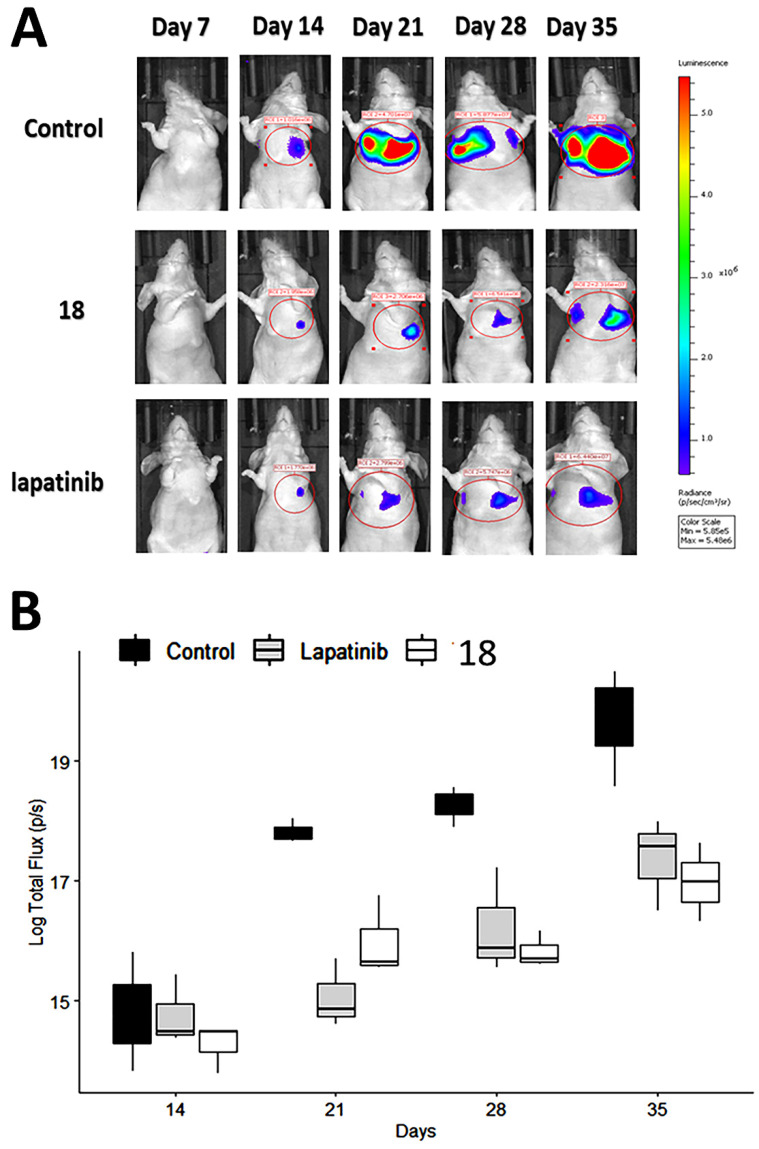
Therapeutic effect of **18** in NSCLC mice model. NSCLC tumors were induced in mice using luciferase transfected A549 cells. Mice were imaged using an *in vivo* imaging instrument after injection of luciferin for tumor growth was monitored. Compound **18** was injected via tail vein, and lapatinib (via intraperitoneal injection) was used as a positive control. **A)** Representative images of tumor growth in mice. Image was processed using software, and ROIs were drawn on the luciferase intensity to quantify the intensity. **B)** Plot of log transformed total flux representing the tumor volume vs. time in days using box and whisker plot. The total flux (p/s) values were log transformed to meet assumptions of an ANOVA. Compound **18** inhibits the tumor growth in lungs significantly compared to control.

Having shown the antiproliferative effects of **18** on A549 cells *in vitro*, we next determined whether these *in vitro* effects correlate with the *in vivo* activity of **18** on pulmonary tumor colonies using an experimental metastasis lung cancer mouse model 57. Although, we have carried out stability studies of compound **18** as possible oral administration, as a proof-of-concept, we carried out *in vivo* studies with IV injection of compound **18** for therapeutic study. A549-Red-FLuc Bioware® Brite Cell Line was injected into the tail vein of mice. The study was carried out with a small group of animals (N = 4/group). The NSCLC cell line A549-Luc is known to be KRAS mutant and EGFR wild-type 45, 58. Each animal was injected with D-luciferin at a dose of 150 mg/Kg intraperitoneally. Using whole-body bioluminescence imaging, tumor growth was monitored by imaging under anesthesia using an IVIS (PerkinElmer) instrument. After a week of cell injection via IV, mice bearing tumors in the lungs were divided into three groups and injected intravenously with either vehicle, lapatinib (10 mg/kg), or **18** (6 mg/kg) in 100 μL prepared in PBS twice a week. This dosage was based on our previous work on **18** in a HER2+ breast cancer mouse model 20 as well as pharmacokinetic studies. The terminal half-life of compound **18** determined was 46 h, and we chose double the half-life time for dosing to maintain the concentration of a drug above IC_50_ also to avoid drug accumulation over time (Supporting Information **Fig. S8**). As shown in **Fig. 5A**, after two weeks, the rate of tumor growth, indicated by total flux (p/s), decreased in mice treated with **18** (6 mg/kg) and mice treated with lapatinib (10 mg/kg) compared with mice injected with vehicle. The total flux (p/s) values were log-transformed to meet the assumptions of an ANOVA. A two-way analysis of variance was carried out with days and treatment as the two factors. Results of the pairwise test (Tukey's honestly significant difference (HSD)) showed that the significant interaction was between lapatinib and **18** treatment. There was no interaction among factors for **18** and lapatinib treatment and control suggesting that a clear statistical difference between **18** treatment and control groups for the 21, 28 and 35 days, *P* = 0.006, *P* = 0.004, *P* = 0.001, respectively (**Fig. 5B**).

### Compound 18 inhibits the phosphorylation of HER2 *in vivo*

**Figure 6:**
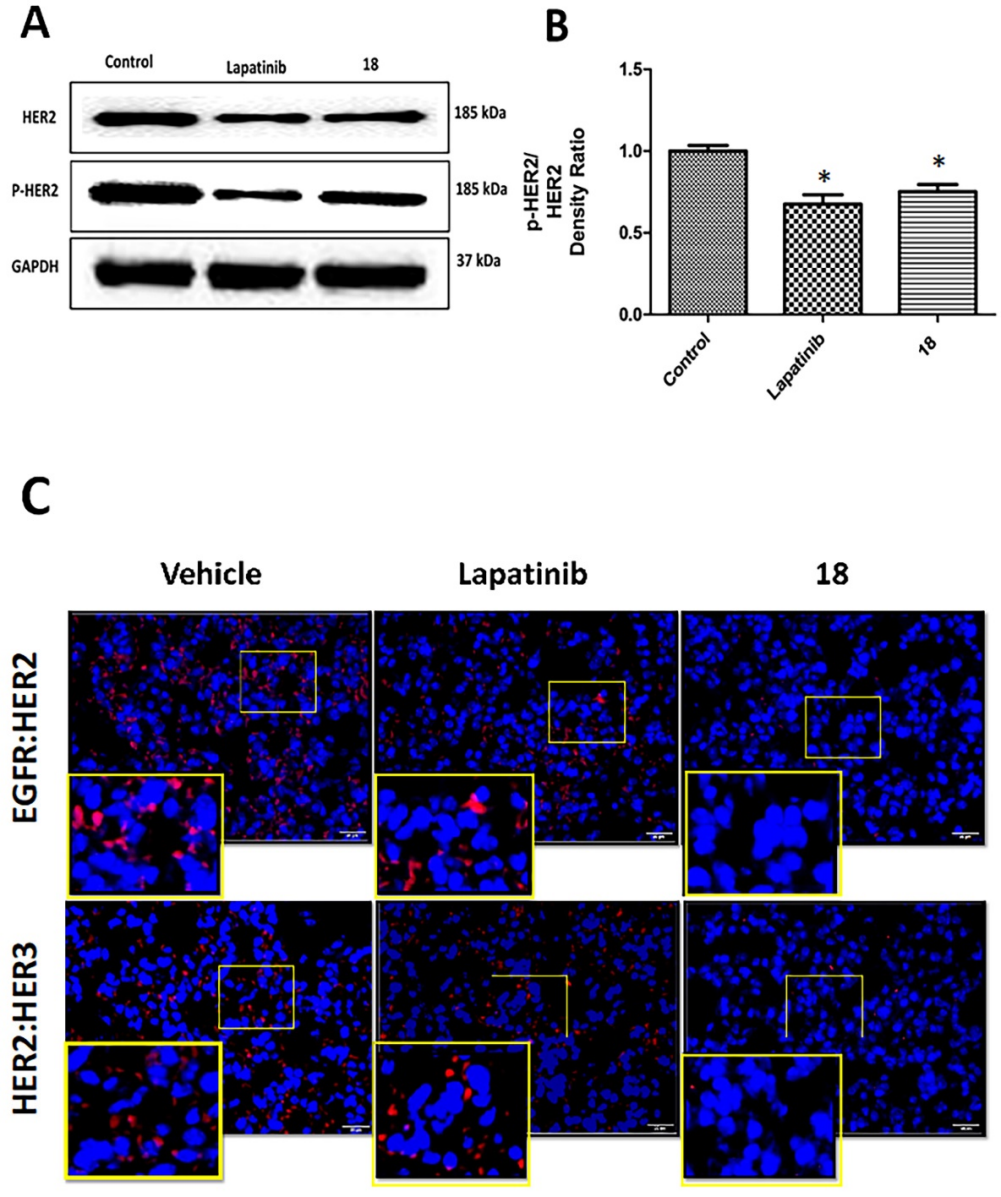
Effect of **18** on phosphorylation of HER2 from lung tumor tissue sections of mice harvested at 35^th^ day. Frozen tumors were processed and Western blot was carried out. (Blots were cropped and presented). Quantification of signal intensities were done by ImageJ). **A)** Western blot images of HER-2, p-HER-2 and GAPDH. **B)** Quantitative analysis of Western blot images indicating reduction in phosphorylation of p-HER2 by **18** and lapatinib (10 mg/kg) compared to the control. **C)** Inhibition of EGFR dimerization in NSCLC tissues from mice by **18** (6 mg/kg) using PLA. Representative tissue sections from lungs of mice from different group of animals were subjected to PLA. Red fluorescence indicates dimerization of EGFR:HER2 (top panel) and HER2:HER3(bottom panel). Expanded regions are shown as insets. Note the reduction in the number of red fluorescence dots in **18**-treated lung tissue. Magnification 40×, size of scale bar 20 μm. Nuclei were stained with DAPI.

Tissue sections of lung tumors were subjected to Western blot to evaluate the phosphorylation of HER2 protein. Images of Western blots indicated a reduction in the phosphorylation of HER2 bands compared to control. Compound **18** and lapatinib inhibited HER2 phosphorylation to a significant extent compared to the control (**Figs 6AB**). Thus, **18** that binds to the extracellular domain inhibits HER2 phosphorylation and, hence, cell signaling in HER2-overexpressing NSCLC *in vivo.*

### Compound 18 inhibits EGFR dimerization *in vivo*

Tissue sections of lung tumor samples were subjected to proximity ligation assay (PLA) to evaluate EGFR dimerization inhibition by **18** *in vivo*. In PLA assay, if the two proteins are in proximity and the distance between the proteins is ≤ 16 nm, those proteins can be targeted with primary and secondary antibodies that are tagged with DNA probes. The probes can be detected with fluorescent tags that specifically bind to the probes. Thus, if the two proteins are in proximity, PLA will indicate a red fluorescence. When the number of EGFR dimers (EGFR:HER2, HER2:HER3) decreases due to inhibition, the fluorescence will decrease (λem 624 nm; Texas Red) 35, 36. Tissue sections from lung cancer and control groups were treated with EGFR, HER2, and HER3 antibodies, and PLA was carried out. Tissue sections from the vehicle control group showed the numbers of red fluorescence dots, indicating EGFR: HER2 and HER2:HER3 dimerization. Sections of tissues that were treated with **18** showed a reduction in red fluorescence, indicating inhibition of EGFR: HER2 and HER2:HER3 dimerization (**Fig. 6C**). These results clearly indicate that inhibition of dimerization prevents signaling for cell growth and, therefore, is related to the reduction in tumor growth rate observed in the treated mouse model.

### Histopathology Studies

**Figure 7:**
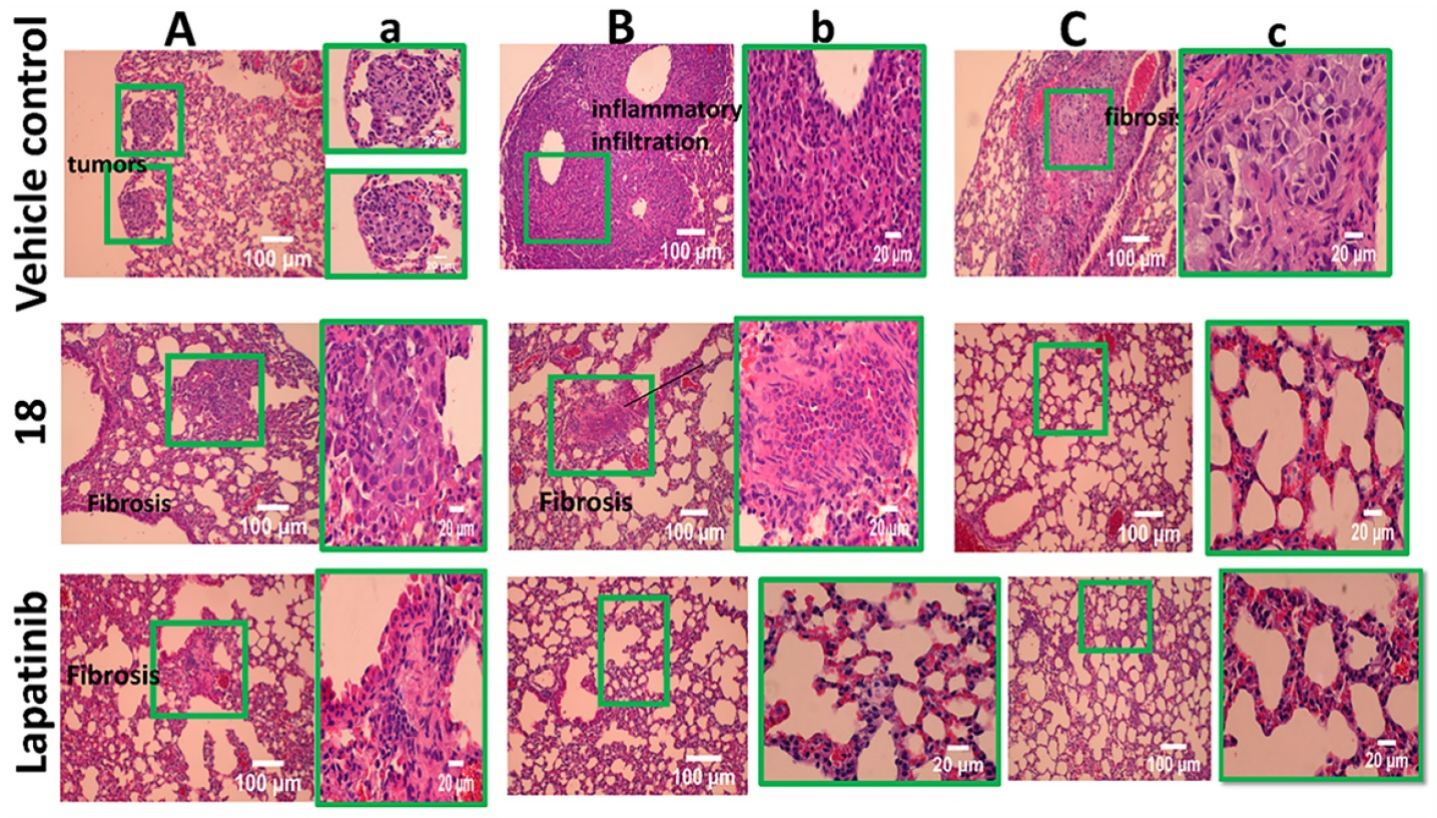
H&E stained tissue sections of lung harvested at 35^th^ day from **18** (6 mg/kg) treated and untreated mice along with control lapatinib-treated. **A, B and C** represent different sections of lung and tumors. a,b, and c are expanded regions shown in the box.

**Figure 8:**
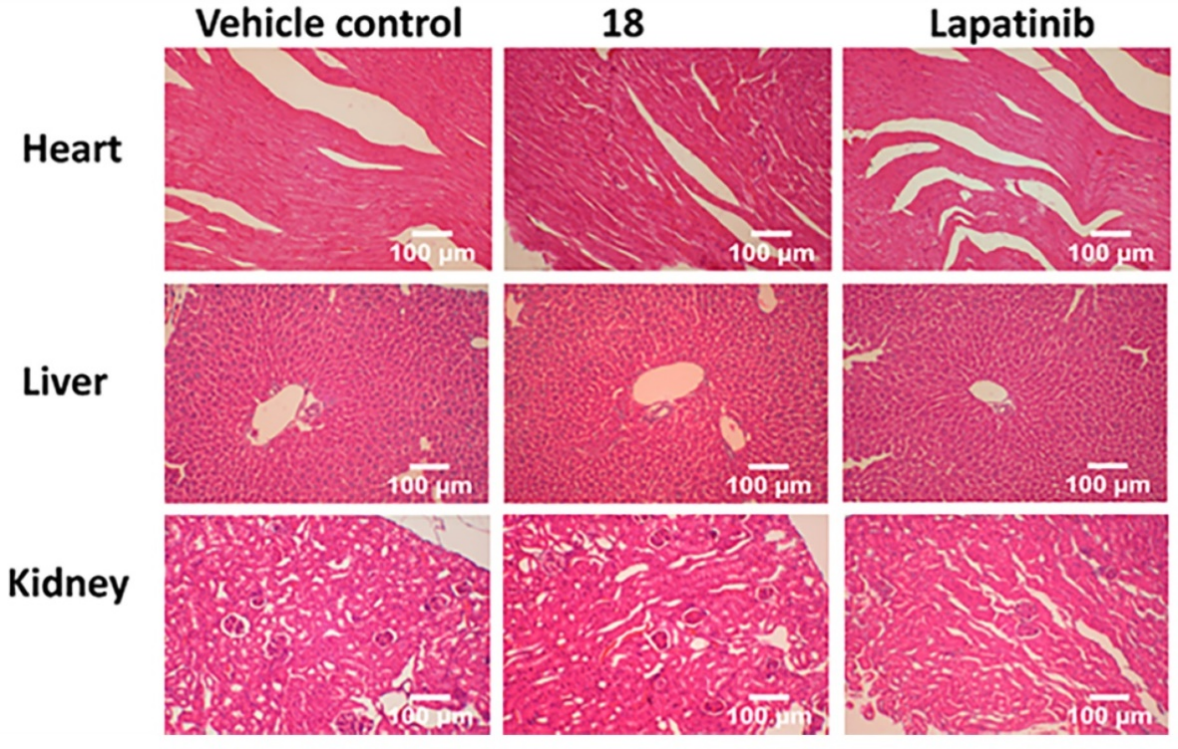
H&E staining of sections of different organs from mice. Tissue section analysis indicated that there was no toxicity to organs in mice at 6 mg/kg dose.

To gain further insight into the therapeutic effect of the treatments on lung tumors, hematoxylin and eosin (H&E)-stained cross-sections of lungs with tumors were studied. H&E staining of histological sections of lungs from mice without treatment (vehicle control) showed the presence of several well-differentiated tumors, inflammatory infiltration, alveolar septal hemorrhage, and congestion (**Fig. 7**). Lung tissue from mice treated with **18** (6 mg/kg) and lapatinib (10 mg/kg) showed remarkably fewer tumors and less inflammatory infiltration compared to those treated with vehicle. To evaluate whether **18** exhibits any toxicity at the dosage studied, tissues of organs such as heart, kidney, and liver were analyzed. Images of H&E-stained organs slices, including heart, liver, lung, and kidney, revealed no significant tissue damage or toxic effects to these organs in either the control group or treated groups (**Fig. 8**).

## Discussion

EGFR family proteins undergo homo- and heterodimerization to generate the signal for cell growth. Clinical studies have shown that HER2 overexpression varies from 3-38% in NSCLC based on the method used for the detection of HER2 7. However, when EGFR and HER2 are both overexpressed in NSCLC, the survival rate is poor. Thus, it could be useful to target a group of individuals who have both EGFR and HER2 overexpression in NSCLC. Our strategy was to minimize the heterodimerization of HER2 receptor and inhibit the downstream signaling pathways (RAS-RAF-MEK-ERK MAPK and AKT-PI3K-mTOR) for cell proliferation, survival, and tumor growth (**Fig. 1**). We have designed a novel peptidomimetic that targets domain IV of HER2 protein and inhibits the dimerization of HER2 with EGFR and HER3. We have shown that **18** is highly specific for HER2- positive cancer cell lines in exhibiting antiproliferative activity 20. For developing non-small cell lung cancer, we used the experimental metastasis lung cancer model. Before going for *in vivo* studies, we did preliminary *in vitro* studies, including antiproliferative evaluation and stability assessment. Compound **18** was evaluated for its antiproliferative activity against A549, adenocarcinoma human alveolar basal epithelial cell line, which overexpresses HER2 and possesses wild type EGFR and KRAS mutation. KRAS mutation is present in about 30% of adenocarcinoma 43, 44 which makes A549 lung cancer model good for our experiments because we would be directly targeting EGFR family and as the peptidomimetic would be inhibiting the proliferation of A549, which means KRAS is also indirectly affected that comprises 30% of adenocarcinoma. The antiproliferative activity showed compound **18** was potent with an IC_50_ value in lower micromolar (0.868±0.032 µM), whereas, as reported in our previous publication, for Calu-3 cell lines that overexpress HER2 IC_50_ was 0.018 ± 0.013 μM and in HER2 positive breast cancer cell lines BT-474 and SKBR-3, IC_50_ was 0.197 ± 0.055 and 0.194 ± 0.046 μM respectively. For MCF-7 cell lines that do not overexpress HER2, IC_50_ was >50 μM 20. The difference observed in IC_50_ value for these cell lines that express HER2 may be due to the difference in the overexpression of HER2 in those cell lines, which also shows that compound **18** is specific towards HER2 receptors 59. These results suggest that the pharmacological action of **18** depends on the dimerization of HER2 with other receptors in cancer cell lines.

HER2 is known to be a dimerization partner for other EGFR receptors, and HER2 activation is also known in EGFR overexpressed, or EGFR mutated NSCLC. Thus, **18** could be useful to target a group of individuals who have both EGFR and HER2 overexpression in NSCLC. A549 cells have HER2 overexpression, *EGFR* wild type, and *KRAS* mutation. Studies have suggested that NSCLC cells with KRAS mutation can be EGFR/HER dependent and EGFR/HER independent pathways. Umelo et al. 60 suggested that upstream inhibition of the EGFR/HER receptors may be effective in treating a subset of KRAS mutant lung cancers. In clinical trials of patients with NSCLC and *KRAS* mutations, data are conflicting regarding the effect of KRAS mutations and treatment outcomes. It was reported that different KRAS mutations could activate distinct signaling pathways. NSCLC cell lines with mutant KRAS have activated phosphatidylinositol 3-kinase (PI3K) and mitogen-activated protein/extracellular signal-regulated kinase (MEK) signaling 61. Compound 18 inhibits EGFR:HER2 and HER2:HER3 dimers, and hence both PI3K and MAPK pathway may be inhibited. Thus, even if KRAS mutation is there, compound **18** could block both pathways activated by a particular type of mutation of KRAS and hence reduce the signaling for cell growth. This might explain why compound **18** is effective in the cancer model of A549 cells.

HER2 is a major proliferative driver that activates downstream signaling of EGFR, such as phosphatidylinositol-3 kinase (PI3K/Akt) and MEK-ERK. To investigate the effect of **18** on downstream signaling, HER2 and Akt phosphorylation were evaluated. Compound **18** is known to target the extracellular domain of HER2 (**Fig. 1BC**). Binding to the extracellular domain of HER2 will also affect the intracellular signaling as **18** inhibits the dimerization of the extracellular domain (ECD) of EGFRs and, in turn, inhibits phosphorylation of intracellular domains. Indeed **18** reduces the phosphorylation of HER2 and Akt, as shown by Western blot (**Fig. 2ABC**). Compared to lapatinib, treatment with **18** showed less reduction in phosphorylation of HER2 and Akt. Lapatinib is a direct inhibitor of HER2 kinase 32, 62, whereas treatment with **18** inhibits dimerization of the extracellular domain that leads to inhibition of phosphorylation of downstream signaling proteins (**Fig. 1C**). In addition, the use of peptidomimetic targeting the extracellular domain can be beneficial over tyrosine kinase inhibitors (TKIs) since the clinical benefits of TKI is limited as TKI develops resistance within few years because of the inevitable activating mutations in the kinase domain of HER2 63, 64.

Stability assessment of compound **18** was performed under different conditions. Compound **18** is a cyclic peptidomimetic, and hence less susceptible to enzymatic degradation by protease 65. HPLC and mass spectrometry analysis showed that compound **18** is stable when incubated from 25°C to 80 °C. Circular dichroism analysis further proved that there was no change in the backbone conformation of compound **18** up to 80°C. Accelerated stability of compound under harsh conditions such as extreme pH and high temperature provides information about long term stability, so pH-dependent stability assessment was done for 30 days. pH stability study showed that compound **18** is stable at pH 7.5, but it slowly degrades at pH 1. There is a significant decrease in the concentration of compound **18** only after 4 days at pH 1, which means it is stable in the stomach if given orally since the residence time of orally active drugs.

To evaluate the possibility of oral administration, the stability of compound **18** was assessed *in vitro* in pooled human liver microsomes, simulated gastric, and simulated intestinal fluid. Human liver microsomes contain a wide variety of drug-metabolizing enzymes and are commonly used to support *in vitro* ADME studies and to examine the potential for the first-pass metabolism of orally administered drugs. When compound **18** was incubated with pooled human liver microsomes in the presence of cofactor NADPH, analysis by HPLC and mass spectrometry revealed no significant degradation up to 6 h. This shows the possibility of limited first-pass metabolism for compound **18**. As the majority of liver xenobiotic metabolic enzymes are cytochromes and carryout oxidation-reduction and hydroxylation mechanisms, we do not expect peptide bond metabolism in liver microsomes but oxidation and reduction of side chains only 66, 67. However, the amino acids in **18** might not be a suitable substrate for the enzymes in liver microsomes for metabolism; therefore, **18** is stable in liver microsomes. During oral drug delivery, degradation of peptide drugs is common due to the highly acidic condition of the stomach together with gastric-secreted enzyme pepsin. When compound **18** was incubated with simulated gastric fluid with pepsin, there was no significant degradation until 6 h. Enzymes secreted in the intestine, i.e., trypsin, chymotrypsin, elastase, and brush-border membrane-bound enzymes carboxypeptidases A and B can also cause hydrolysis of peptide and lead to its degradation and loss of activity. To evaluate the stability of compound **18** in intestinal fluid, it was incubated with simulated intestinal fluid for 12 hours. HPLC analysis showed that compound **18** slowly degrades in simulated intestinal fluid. We evaluated pharmacokinetics properties of **18** in nude mice. The terminal half-life of compound **18** following 6 mg/kg and 4 mg/kg IV dose administration was predicted to be around 46 and 42 hours, respectively.

Compound **18** is a peptidomimetic with the modified backbone as well as a side with 3-amino-3-(1-napthyl propionic acid) (Anapa) functional group. Since there are two Anapa functional groups in **18** and is cyclized via a peptide bond, we anticipated that **18** is stable *in vivo* for its pharmacological action. The measurement of plasma drug concentrations doesn't provide clear insight into the relationship between the free and the plasma-protein-bound fractions of drugs. To understand how compound **18** binds to the Human serum albumin, SPR analysis, and molecular docking was performed. The equilibrium dissociation constant (K_d_) calculated was 190 µM indicating 78% of compound **18** bound to HSA (nearly 22% of compound **18** in free form). Studies related to serum protein binding of drugs suggests that drugs that exhibit 11 to 20% of the free drug are favorable property of the drugs, particularly for peptide-based drugs to have relatively long *in vivo* half-life 68, 69. Here we considered only human serum albumin, and not other proteins that bind to drugs such as α-acid glycoprotein, since human serum albumin is the most abundant protein in blood plasma. It has been reported in the literature that site I ligands appear to be dicarboxylic acids and/or bulky heterocyclic molecules with a negative charge localized in the middle of the molecule. Molecular docking showed compound **18** binds to drug binding site 1. Binding of compound **18** to site I might be attributable to Anapa and Aspartic acid residues of compound **18** 70. Molecular modeling also revealed the binding energy of compound **18** with HSA to be -7.67 Kcal/mol. From SPR and molecular docking analysis, we can predict that compound **18** binds weakly to serum albumin.

The longer half-life *in vivo* might be due to rapid distribution and accumulation of compound **18** in the tissue compartment, but further experiments are needed to be done. Results from pharmacokinetic studies indicated longer half-life of compound **18** via iv dosing, the anti-tumor activity of compound **18** was evaluated in experimental metastasis lung cancer mice model via iv dosing as a proof-of-concept. The terminal half-life of compound **18** following 6 mg/kg and 4 mg/kg IV single-dose administration was estimated to be around 46 hours and 42 hours, respectively (**Fig. 4AB**). Distribution half-life, V_d central_, V_d peripheral_, AUC_ (0-∞)_, and clearance were estimated at around 0.8 h, 11 ml, 17 ml, 459 μg/ml∙h, and 0.26ml/hr respectively. Generally, acceptable PK parameters for drugs are described by Motty 71, and PK studies of peptides are reported by others 72. The relatively long half-life of compound **18** in plasma is advantageous, because longer half-lives normally translate to less-frequent dosing requirements (and of course, the half-life must be long enough in the first place to allow efficacious concentrations to be achieved in target tissues at steady state, even more essential with routes of dosing other than IV). Considering that compound **18** is a peptidomimetic, and that the IC_50_ value was seen to be in the nanomolar concentration range for HER2-positive lung cancer cells, the estimated pharmacokinetic half-life should be long enough to enable efficacious exposure levels in tissues provided a suitable dosing regimen were designed. Based on the molecular mechanism, we would want the trough levels (lowest level before each following dose) to be well above the IC_50_ observed for the corresponding cells, at least most of the time. We have calculated the trough levels of the drug *in vivo* in plasma and plotted a graph. We have also calculated the fraction of drug unbound to the serum. Steady-state trough levels remain above the IC_50_ value for cell proliferation observed in cultured cells (Supporting Information**Figs. S6-S8).**

Tail vein injection of A549 cells is known to colonize in the lungs of the mice and is a very good model to study NSCLC tumors in mice 73, 74. The growth of tumors in the lung can be measured by imaging of luminescence from luciferin without sacrificing the animal. It has been reported that the photon flux from tumors is directly proportional to the number of light-emitting cells that express luciferase, and the signal can be measured to monitor tumor growth and development 75. Bioluminescence intensity in the result indicated that the treatment of **18** via intravenous administration help to reduce the NSCLC tumor growth in the lungs in a mice model. Analysis of tissue sections from animals that were treated with compound at 6 mg/kg suggests that **18** did not exhibit any toxic effect on organs such as heart, kidney, and liver in the model animal. Although we have not carried detailed toxicity studies to determine the maximum tolerated dose (MTD) of compound **18** in an animal model, based on our therapeutic dose and histopathology analysis of organs, the relatively long half-life of **18** and three weeks of treatments did not cause any toxic effect on animals.

Dimerization of EGFR in lung tumor suggested that these lung tumor tissues exhibit EGFR: HER2 and HER2:HER3 dimers and **18** were able to inhibit both the dimers as shown by PLA (**Fig. 6C**) on lung tumor tissue. Thus, **18** is a dual inhibitor of EGFR dimers in lung tissue. Compound **18** can also be used in HER2 overexpression, mutation, and amplification since these processes ultimately result in HER2 homo and heterodimerization, and our peptidomimetic are designed to disrupt HER2 dimerization by binding to ECD. Although, HER2 overexpression in clinical studies is only 3-38%, overexpression is not the only criteria for targeting with **18**. The dimerization of HER2 with other EGFR receptors is an important criterion. Compound **18** also inhibits the dimerization of HER2 with its dimerization partner that further inhibits cell proliferation. HER2 does not have any known ligand and is activated by dimerization with other receptors such as EGFR and HER3 76. Spontaneous dimerization of HER2 occurs via gene amplification or kinase activation by EGFR or HER3 77. *HER2* amplification is considered an alternative mechanism for the development of resistance to EGFR-targeted TKI therapy 78. Published data indicate that the HER2/HER3 signaling pathways play an important role both in NSCLC tumors resistant to inhibitors of EGFR (cetuximab and erlotinib) 78-81 and in NSCLC cancers driven by activating mutations in *HER2* (4% of NCSLC patients) 82. Spontaneous dimerization of HER2 occurs via gene amplification or kinase activation by EGFR or HER3. Furthermore, HER2 dimerization with other EGFRs is known to be a driver for NSCLC. HER2 dimerization with EGFR and HER3 is known to be persistent in EGFR kinase mutated NSCLC 76, 77, 83, 84. Thus, inhibition of both EGFR:HER2 and HER2:HER3 is known to be advantageous in the treatment of NSCLC. Dimerization of EGFRs occur via extracellular domain II and IV. Compound **18** is known to bind to ECD of domain IV of HER2 and inhibit the dimerization of HER2 with EGFR and HER3. This inhibition is known to inhibit phosphorylation of the kinase domain and downstream signaling such as PI3K and Akt and MAPK pathway and hence signaling for cell growth. Using SPR, PLA and Western blot assay as well as cell proliferation activity, we have shown that compound **18** binds to domain IV of HER2, inhibits dimerization, HER2 kinase phosphorylation, downstream signaling Akt and hence cell proliferation in lung cancer cell lines. In mice model, the tissue sections from lung tumors were evaluated for HER2 expression (Supporting Information**Fig. S9**). These tissue samples exhibit HER2 overexpression compared to normal lung tissue. Our previous experiments have shown that compound **18** does not affect the HER2 expression in tumor tissue, rather inhibit the HER2 dimerization 20 (Supporting Information**Fig. S10**). Thus, **18** that inhibits the dimerization might be useful for NSCLC that have EGFR resistance but express EGFR dimers.

## Conclusions

In summary, HER2 overexpression and mutations are present in a subset of lung adenocarcinomas. Patients with HER2 alterations have worse survival outcomes as compared to patients without these alterations. Research into targeted therapies that target HER2 in lung cancers has the potential to improve outcomes in a subset of patients with HER2 alterations. In this study, a peptidomimetic, compound **18**, is designed that binds to domain IV of the HER2 receptor, disrupts the homo/heterodimerization of HER2, and inhibits downstream signaling for cellular proliferation and growth, hence, can be used as a potential therapeutic agent for the treatment of NSCLC.

## Supplementary Material

Supplementary figures and tables.

## Abbreviations

ADME: Absorption, Distribution, Metabolism, and Excretion
AIC: Akaike's information criterion
ALK: anaplastic lymphoma kinase
AUC: area under the curve
CHCA: α-cyano-4-hydroxycinnamic acid
DAPI: 4′,6-diamidino-2-phenylindole
DMSO: dimethylsulfoxide
ECD: extracellular domain
EGFR: epidermal growth factor receptor
FDA: food and drug administration
H&E: hematoxylin and eosin
HSD: honestly significant difference
HSA: human serum albumin
HER2: human epidermal growth factor receptor 2
KRAS: Kirsten ras sarcoma
LGA: Lamarckian genetic algorithm
MALDI-TOF MS: Matrix-Assisted Laser Desorption/Ionization-Time Of Flight mass spectrometry
MEK: mitogen-activated protein/extracellular signal-regulated kinase
MTD: maximum tolerated dose
NADPH: Nicotinamide adenine dinucleotide phosphate
NSCLC: non-small cell lung cancer
PBS: phosphate buffered saline
PD-1-PD-L1: programmed cell death protein 1-programmed death-ligand 1
PI3K: phosphatidylinositol 3-kinase
ROI: regions of interest
SC: Schwarz criterion
SEM: standard error of the mean
SCLC: small cell lung cancer
SPR: Surface plasmon resonance
TFA: trifluoroacetic acid
TKI: tyrosine kinase inhibitor
VEGFR: vascular endothelial growth factor receptor

## References

[1] Siegel RL, Miller KD, Jemal A (2018). Cancer statistics, 2018. CA Cancer J Clin.

[2] Herbst RS, Morgensztern D, Boshoff C (2018). The biology and management of non-small cell lung cancer. Nature.

[3] Kris MG, Johnson BE, Berry LD, Kwiatkowski DJ, Iafrate AJ, Wistuba, II (2014). Using multiplexed assays of oncogenic drivers in lung cancers to select targeted drugs. JAMA.

[4] Silva APS, Coelho PV, Anazetti M, Simioni PU (2016). Targeted therapies for the treatment of non-small-cell lung cancer: Monoclonal antibodies and biological inhibitors. Human vaccines & immunotherapeutics.

[5] Yousefi H, Yuan J, Keshavarz-Fathi M, Murphy JF, Rezaei N (2017). Immunotherapy of cancers comes of age. Expert Rev Clin Immunol.

[6] Melosky B, Juergens R, Hirsh V, McLeod D, Leighl N, Tsao MS (2019). Amplifying Outcomes: Checkpoint Inhibitor Combinations in First-Line Non-Small Cell Lung Cancer. Oncologist.

[7] Kim EK, Kim KA, Lee CY, Shim HS (2017). The frequency and clinical impact of HER2 alterations in lung adenocarcinoma. PLoS One.

[8] Ricciardi GR, Russo A, Franchina T, Ferraro G, Zanghi M, Picone A (2014). NSCLC and HER2: between lights and shadows. J Thorac Oncol.

[9] Oh DY, Bang YJ (2020). HER2-targeted therapies - a role beyond breast cancer. Nat Rev Clin Oncol.

[10] Sapino A, Goia M, Recupero D, Marchiò C (2013). Current Challenges for HER2 Testing in Diagnostic Pathology: State of the Art and Controversial Issues. Frontiers in oncology.

[11] Battino M, Giampieri F, Pistollato F, Sureda A, de Oliveira MR, Pittalà V (2018). Nrf2 as regulator of innate immunity: A molecular Swiss army knife!. Biotechnology Advances.

[12] Lara PN Jr, Laptalo L, Longmate J, Lau DH, Gandour-Edwards R, Gumerlock PH (2004). Trastuzumab plus docetaxel in HER2/neu-positive non-small-cell lung cancer: a California Cancer Consortium screening and phase II trial. Clin Lung Cancer.

[13] Geyer CE, Forster J, Lindquist D, Chan S, Romieu CG, Pienkowski T (2006). Lapatinib plus capecitabine for HER2-positive advanced breast cancer. N Engl J Med.

[14] Guarneri V, Frassoldati A, Bottini A, Cagossi K, Bisagni G, Sarti S (2012). Preoperative chemotherapy plus trastuzumab, lapatinib, or both in human epidermal growth factor receptor 2-positive operable breast cancer: results of the randomized phase II CHER-LOB study. J Clin Oncol.

[15] Hirsch FR, Scagliotti GV, Mulshine JL, Kwon R, Curran WJ Jr, Wu YL (2017). Lung cancer: current therapies and new targeted treatments. Lancet.

[16] Camidge DR, Pao W, Sequist LV (2014). Acquired resistance to TKIs in solid tumours: learning from lung cancer. Nat Rev Clin Oncol.

[17] Valley CC, Arndt-Jovin DJ, Karedla N, Steinkamp MP, Chizhik AI, Hlavacek WS (2015). Enhanced dimerization drives ligand-independent activity of mutant epidermal growth factor receptor in lung cancer. Mol Biol Cell.

[18] Casalini P, Iorio MV, Galmozzi E, Menard S (2004). Role of HER receptors family in development and differentiation. J Cell Physiol.

[19] Wee P, Wang Z (2017). Epidermal Growth Factor Receptor Cell Proliferation Signaling Pathways. Cancers.

[20] Kanthala SP, Liu YY, Singh S, Sable R, Pallerla S, Jois SD (2017). A peptidomimetic with a chiral switch is an inhibitor of epidermal growth factor receptor heterodimerization. Oncotarget.

[21] Qiu Y, Taichi M, Wei N, Yang H, Luo KQ, Tam JP (2017). An orally active bradykinin B1 receptor antagonist engineered as a bifunctional chimera of sunflower trypsin inhibitor. J. Med. Chem.

[22] Harding FA, Stickler MM, Razo J, DuBridge RB (2010). The immunogenicity of humanized and fully human antibodies: residual immunogenicity resides in the CDR regions. mAbs.

[23] Esserman LJ, Demichele A (2014). Accelerated Approval for Pertuzumab in the Neoadjuvant Setting: Winds of Change?. Clin Cancer Res.

[24] Franklin MC, Carey KD, Vajdos FF, Leahy DJ, de Vos AM, Sliwkowski MX (2004). Insights into ErbB signaling from the structure of the ErbB2-pertuzumab complex. Cancer Cell.

[25] Barthelemy P, Leblanc J, Goldbarg V, Wendling F, Kurtz JE (2014). Pertuzumab: development beyond breast cancer. Anticancer research.

[26] Hughes B, Mileshkin L, Townley P, Gitlitz B, Eaton K, Mitchell P (2014). Pertuzumab and erlotinib in patients with relapsed non-small cell lung cancer: a phase II study using 18F-fluorodeoxyglucose positron emission tomography/computed tomography imaging. The oncologist.

[27] Scheuer W, Friess T, Burtscher H, Bossenmaier B, Endl J, Hasmann M (2009). Strongly enhanced antitumor activity of trastuzumab and pertuzumab combination treatment on HER2-positive human xenograft tumor models. Cancer research.

[28] Felip E, Ranson M, Cedres S, Dean E, Brewster M, Martinez P (2012). A phase Ib, dose-finding study of erlotinib in combination with a fixed dose of pertuzumab in patients with advanced non-small-cell lung cancer. Clin Lung Cancer.

[29] Nami B, Maadi H, Wang Z (2018). Mechanisms Underlying the Action and Synergism of Trastuzumab and Pertuzumab in Targeting HER2-Positive Breast Cancer. Cancers (Basel).

[30] Kanthala S, Banappagari S, Gokhale A, Liu Y-Y, Xin G, Zhao Y (2015). Novel Peptidomimetics for Inhibition of HER2:HER3 Heterodimerization in HER2-Positive Breast Cancer. Chemical biology & drug design.

[31] Tolliday N (2010). High-throughput assessment of Mammalian cell viability by determination of adenosine triphosphate levels. Curr Protoc Chem Biol.

[32] Diaz R, Nguewa PA, Parrondo R, Perez-Stable C, Manrique I, Redrado M (2010). Antitumor and antiangiogenic effect of the dual EGFR and HER-2 tyrosine kinase inhibitor lapatinib in a lung cancer model. BMC Cancer.

[33] Wang J, Yadav V, Smart AL, Tajiri S, Basit AW (2015). Toward oral delivery of biopharmaceuticals: an assessment of the gastrointestinal stability of 17 peptide drugs. Mol Pharm.

[34] Morris GM, Huey R, Lindstrom W, Sanner MF, Belew RK, Goodsell DS (2009). AutoDock4 and AutoDockTools4: Automated docking with selective receptor flexibility. Journal of computational chemistry.

[35] Soderberg O, Gullberg M, Jarvius M, Ridderstrale K, Leuchowius KJ, Jarvius J (2006). Direct observation of individual endogenous protein complexes *in situ* by proximity ligation. Nat Methods.

[36] Fredriksson S, Gullberg M, Jarvius J, Olsson C, Pietras K, Gustafsdottir SM (2002). Protein detection using proximity-dependent DNA ligation assays. Nature biotechnology.

[37] Banappagari S, Ronald S, Satyanarayanajois SD (2011). Structure-activity relationship of conformationally constrained peptidomimetics for antiproliferative activity in HER2-overexpressing breast cancer cell lines. MedChemComm.

[38] Satyanarayanajois S, Villalba S, Jianchao L, Lin GM (2009). Design, synthesis, and docking studies of peptidomimetics based on HER2-herceptin binding site with potential antiproliferative activity against breast cancer cell lines. Chem Biol Drug Des.

[39] Banappagari S, Ronald S, Satyanarayanajois SD (2010). A conformationally constrained peptidomimetic binds to the extracellular region of HER2 protein. Journal of biomolecular structure & dynamics.

[40] Banappagari S, Corti M, Pincus S, Satyanarayanajois S (2012). Inhibition of protein-protein interaction of HER2-EGFR and HER2-HER3 by a rationally designed peptidomimetic. Journal of biomolecular structure & dynamics.

[41] Kanthala S, Gauthier T, Satyanarayanajois S (2014). Structure-activity relationships of peptidomimetics that inhibit PPI of HER2-HER3. Biopolymers.

[42] Pallerla S, Naik H, Singh S, Gauthier T, Sable R, Jois SD (2018). Design of cyclic and d-amino acids containing peptidomimetics for inhibition of protein-protein interactions of HER2-HER3. J Pept Sci.

[43] Bunn PA, Helfrich B, Soriano AF, Franklin WA, Varella-Garcia M, Hirsch FR (2001). Expression of Her-2/neu in Human Lung Cancer Cell Lines by Immunohistochemistry and Fluorescence *in situ* Hybridization and Its Relationship to *in vitro* Cytotoxicity by Trastuzumab and Chemotherapeutic Agents. Clin Cancer Res.

[44] Yang S, Yu X, Fan Y, Shi X, Jin Y (2018). Clinicopathologic characteristics and survival outcome in patients with advanced lung adenocarcinoma and KRAS mutation. J Cancer.

[45] Suzawa K, Toyooka S, Sakaguchi M, Morita M, Yamamoto H, Tomida S (2016). Antitumor effect of afatinib, as a human epidermal growth factor receptor 2-targeted therapy, in lung cancers harboring HER2 oncogene alterations. Cancer Sci.

[46] Blessy M, Patel RD, Prajapati PN, Agrawal YK (2014). Development of forced degradation and stability indicating studies of drugs-A review. J Pharm Anal.

[47] Fallingborg J (1999). Intraluminal pH of the human gastrointestinal tract. Dan Med Bull.

[48] Knights KM, Stresser DM, Miners JO, Crespi CL *In vitro* drug metabolism using liver microsomes. Current protocols in pharmacology. 2016: 7.8. 1-7.8. 24.

[49] Jenssen H, Aspmo SI (2008). Serum stability of peptides. Methods Mol Biol.

[50] Zhang Y, Huo M, Zhou J, Xie S (2010). PKSolver: An add-in program for pharmacokinetic and pharmacodynamic data analysis in Microsoft Excel. Comput Methods Programs Biomed.

[51] Mohammed E, Naugler C, Far B (2015). Emerging Business Intelligence Framework for a Clinical Laboratory Through Big Data Analytics. Emerging Trends in Computational Biology, Bioinformatics, and Systems Biology.

[52] Howard ML, Hill JJ, Galluppi GR, McLean MA (2010). Plasma protein binding in drug discovery and development. Combinatorial chemistry & high throughput screening.

[53] Otagiri M (2009). Study on binding of drug to serum protein. Yakugaku Zasshi.

[54] Rich RL, Day YS, Morton TA, Myszka DG (2001). High-resolution and high-throughput protocols for measuring drug/human serum albumin interactions using BIACORE. Analytical biochemistry.

[55] Petitpas I, Bhattacharya AA, Twine S, East M, Curry S (2001). Crystal structure analysis of warfarin binding to human serum albumin: anatomy of drug site I. J Biol Chem.

[56] Fasano M, Curry S, Terreno E, Galliano M, Fanali G, Narciso P (2005). The extraordinary ligand binding properties of human serum albumin. IUBMB Life.

[57] Muralidharan R, Babu A, Amreddy N, Srivastava A, Chen A, Zhao YD (2017). Tumor-targeted Nanoparticle Delivery of HuR siRNA Inhibits Lung Tumor Growth *In vitro* and *In vivo* By Disrupting the Oncogenic Activity of the RNA-binding Protein HuR. Mol Cancer Ther.

[58] Ding L, Tian C, Feng S, Fida G, Zhang C, Ma Y (2015). Small sized EGFR1 and HER2 specific bifunctional antibody for targeted cancer therapy. Theranostics.

[59] Cretella D, Saccani F, Quaini F, Frati C, Lagrasta C, Bonelli M (2014). Trastuzumab emtansine is active on HER-2 overexpressing NSCLC cell lines and overcomes gefitinib resistance. Mol Cancer.

[60] Umelo IA, De Wever O, Kronenberger P, Van Deun J, Noor A, Singh K (2015). Combined targeting of EGFR/HER promotes anti-tumor efficacy in subsets of KRAS mutant lung cancer resistant to single EGFR blockade. Oncotarget.

[61] Ihle NT, Byers LA, Kim ES, Saintigny P, Lee JJ, Blumenschein GR (2012). Effect of KRAS oncogene substitutions on protein behavior: implications for signaling and clinical outcome. J Natl Cancer Inst.

[62] Wainberg ZA, Anghel A, Desai AJ, Ayala R, Luo T, Safran B (2010). Lapatinib, a dual EGFR and HER2 kinase inhibitor, selectively inhibits HER2-amplified human gastric cancer cells and is synergistic with trastuzumab *in vitro* and *in vivo*. Clin Cancer Res.

[63] Lin Y, Wang X, Jin H (2014). EGFR-TKI resistance in NSCLC patients: mechanisms and strategies. Am J Cancer Res.

[64] Pillai RN, Behera M, Berry LD, Rossi MR, Kris MG, Johnson BE (2017). HER2 mutations in lung adenocarcinomas: A report from the Lung Cancer Mutation Consortium. Cancer.

[65] Gang D, Kim DW, Park H-S (2018). Cyclic Peptides: Promising Scaffolds for Biopharmaceuticals. Genes (Basel).

[66] Jia L, Liu X (2007). The conduct of drug metabolism studies considered good practice (II): *in vitro* experiments. Curr Drug Metab.

[67] Zvereva I, Semenistaya E, Krotov G, Rodchenkov G (2016). Comparison of various *in vitro* model systems of the metabolism of synthetic doping peptides: Proteolytic enzymes, human blood serum, liver and kidney microsomes and liver S9 fraction. J Proteomics.

[68] Leithold LH, Jiang N, Post J, Ziehm T, Schartmann E, Kutzsche J (2016). Pharmacokinetic Properties of a Novel D-Peptide Developed to be Therapeutically Active Against Toxic beta-Amyloid Oligomers. Pharm Res.

[69] Kratochwil NA, Huber W, Muller F, Kansy M, Gerber PR (2002). Predicting plasma protein binding of drugs: a new approach. Biochem Pharmacol.

[70] Otagiri M (2005). A Molecular Functional Study on the Interactions of Drugs with Plasma Proteins. Drug metabolism and pharmacokinetics.

[71] Sobol M Drug-like properties: Concepts, structure design and methods from ADME to toxicity optimization [Book Review]. Chemistry in Australia. 2018: 28.

[72] Ramanathan-Girish S, McColm J, Clements JM, Taupin P, Barrowcliffe S, Hevizi J (2004). Pharmacokinetics in animals and humans of a first-in-class peptide deformylase inhibitor. Antimicrobial agents and chemotherapy.

[73] Kellar A, Egan C, Morris D (2015). Preclinical Murine Models for Lung Cancer: Clinical Trial Applications. Biomed Res Int.

[74] Zhang X, Liu Y, Peng X, Zeng Y, Li L, Wang J (2018). Influence of the vaccinating density of A549 cells on tumorigenesis and distant organ metastasis in a lung cancer mice model. Cell Mol Biol (Noisy-le-grand).

[75] Lim E, Modi KD, Kim J *In vivo* bioluminescent imaging of mammary tumors using IVIS spectrum. Journal of visualized experiments: JoVE. 2009: 1210.

[76] Mazieres J, Peters S, Lepage B, Cortot AB, Barlesi F, Beau-Faller M (2013). Lung cancer that harbors an HER2 mutation: epidemiologic characteristics and therapeutic perspectives. J Clin Oncol.

[77] Wang SE, Narasanna A, Perez-Torres M, Xiang B, Wu FY, Yang S (2006). HER2 kinase domain mutation results in constitutive phosphorylation and activation of HER2 and EGFR and resistance to EGFR tyrosine kinase inhibitors. Cancer Cell.

[78] Chong CR, Janne PA (2013). The quest to overcome resistance to EGFR-targeted therapies in cancer. Nat Med.

[79] Takezawa K, Pirazzoli V, Arcila ME, Nebhan CA, Song X, de Stanchina E (2012). HER2 amplification: a potential mechanism of acquired resistance to EGFR inhibition in EGFR-mutant lung cancers that lack the second-site EGFRT790M mutation. Cancer Discov.

[80] Peters S, Zimmermann S (2014). Targeted therapy in NSCLC driven by HER2 insertions. Transl Lung Cancer Res.

[81] Iida M, Bahrar H, Brand TM, Pearson HE, Coan JP, Orbuch RA (2016). Targeting the HER Family with Pan-HER Effectively Overcomes Resistance to Cetuximab. Mol Cancer Ther.

[82] Li BT, Ross DS, Aisner DL, Chaft JE, Hsu M, Kako SL (2016). HER2 Amplification and HER2 Mutation Are Distinct Molecular Targets in Lung Cancers. J Thorac Oncol.

[83] Brabender J, Danenberg KD, Metzger R, Schneider PM, Park J, Salonga D (2001). Epidermal growth factor receptor and HER2-neu mRNA expression in non-small cell lung cancer Is correlated with survival. Clin Cancer Res.

[84] Ota K, Harada T, Otsubo K, Fujii A, Tsuchiya Y, Tanaka K (2017). Visualization and quantitation of epidermal growth factor receptor homodimerization and activation with a proximity ligation assay. Oncotarget.

