# Supplementary material for: In vivo studies of a peptidomimetic that targets EGFR dimerization in NSCLC
Source: J Cancer. 2020 Aug 18;11(20):5982–99. doi: 10.7150/jca.46320 (PMC7477407; doi:10.7150/jca.46320)
Supplement: Supplementary file 1 — Supplementary figures and tables. [file jcav11p5982s1.pdf]

In vivo Studies of a Peptidomimetic that Targets EGFR Dimerization in NSCLC

Leeza Shrestha[1], Sitanshu Singh[1], Pravin Parajuli[1], Achyut Dahal[1], George Mattheolabakis[1], Sharon Meyer[1], Joydeep Bhattacharjee[2], Seetharama Jois[1]*

[1]School of Basic Pharmaceutical and Toxicological Sciences, College of Pharmacy, University of Louisiana Monroe, Monroe, LA 71201.

[2]Biology Program, School of Sciences, University of Louisiana Monroe, Monroe, LA 71029.

Leeza

Supporting Information

Table S1. Antiproliferative activity of compound 18 in different cell lines showing selectivity of compound **18** for HER2 positive cancer cell lines.

**Cell line**	**Type/Source**	**Protein expression**	IC_{50} (μM)
Calu-3	NSCLC cell line	HER2 +	0.018 ± 0.013
A549	NSCLC cell line	HER2 +	0.868 ± 0.032
SKBR-3*	Breast Cancer cell line	HER2 +	0.194 ± 0.046
BT-474*	Breast Cancer cell line	HER2 +	0.197 ± 0.055
SKOV-3	Ovarian Cancer cell line	HER2 +	0.853 ± 0.102
MCF-7	Breast Cancer cell line	HER2 -	>50
MCF-10A	Non-cancerous breast cell line	Basal level expression of HER2	40
NCI-H1975	EGFR mutated NSCLC cell line	EGFR overexpressed-mutated	4.85 ± 0.217

*Kanthala et al. *Oncotarget* 8(43):74244-74262.

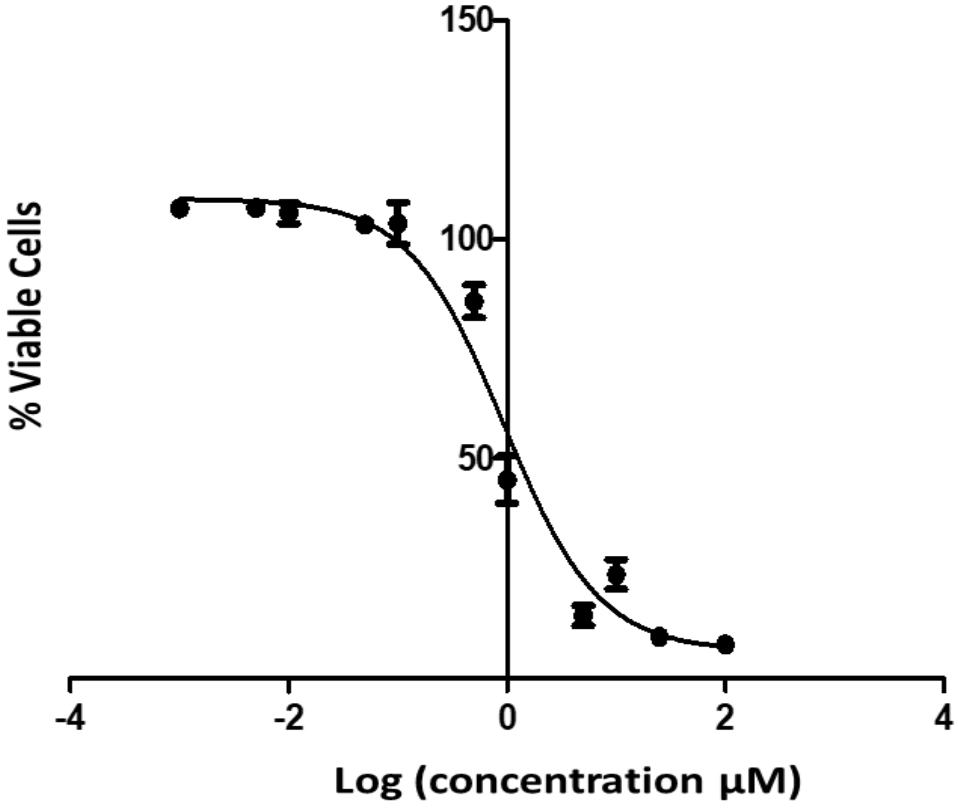

Fig. S1. Response-vs-concentration curve for compound **18** in A549 cell line after incubating 72 h in serum-free medium.

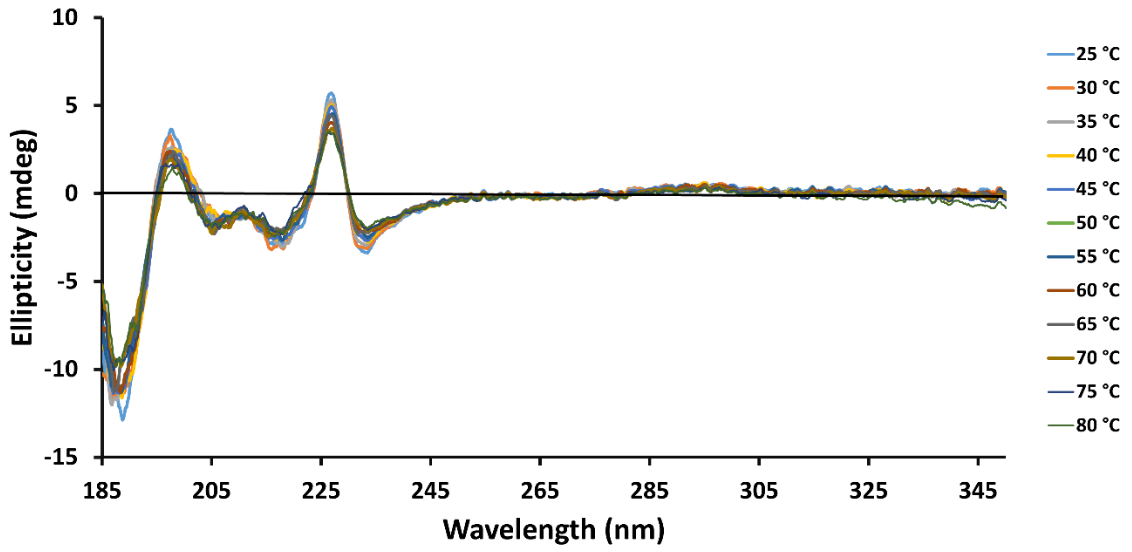

Fig. S2. Overlay of circular dichroism spectra of compound **18** over a temperature range of 0 to 80 °C. For each measurement 4 accumulations were acquired and average spectrum is presented.

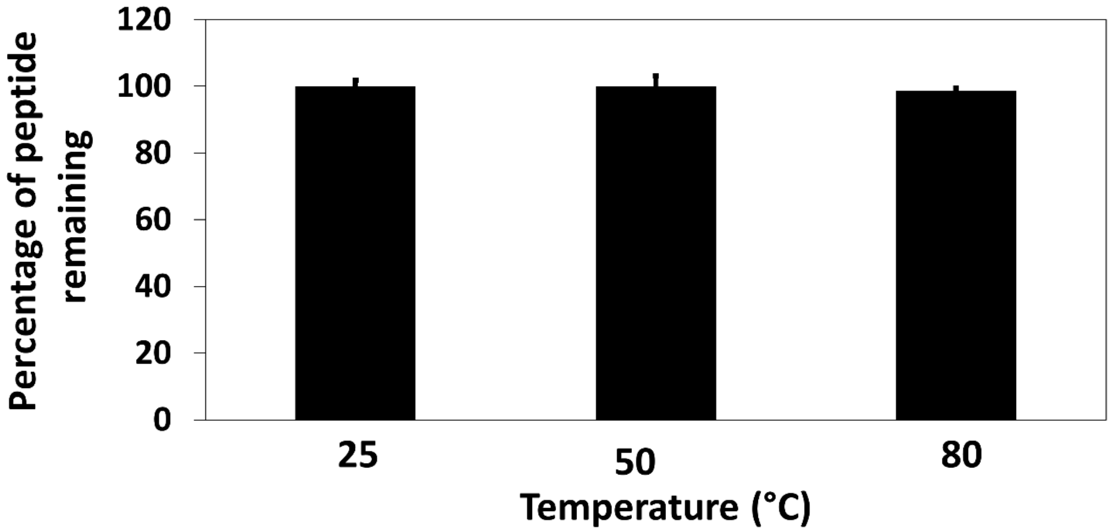

Fig. S3. HPLC analysis of compound **18** incubated at 25, 50 and 80 °C. Peak area calculated at 25 °C was considered as 100% and peaks obtained at 50 and 80 °C were plotted relative to the 25 °C data (taken as 100%). Data from triplicate experiments.

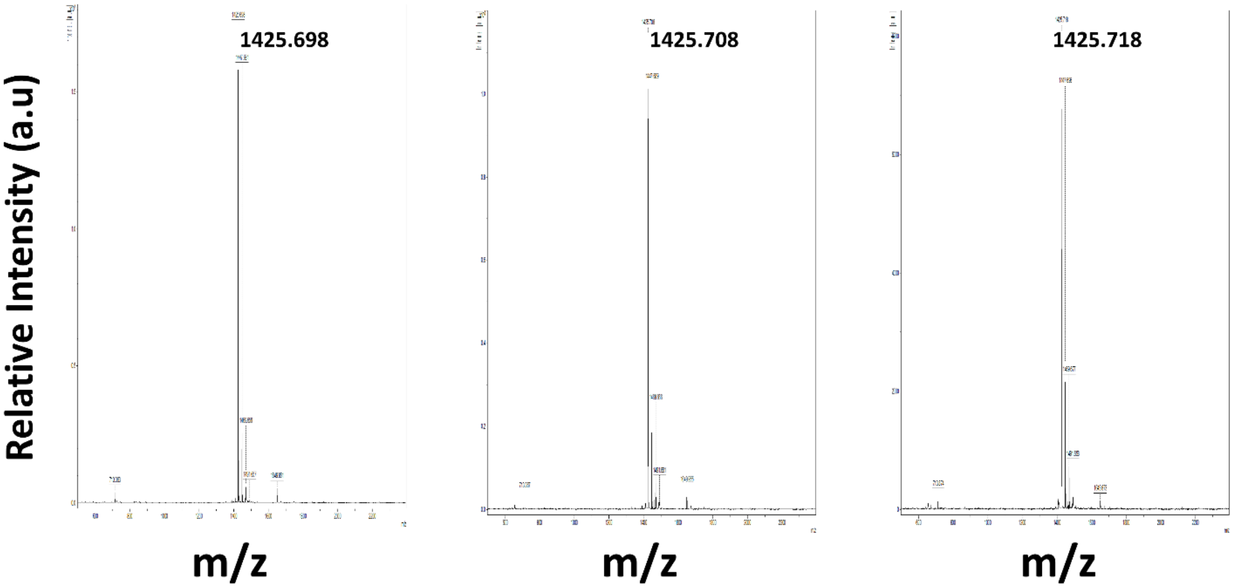

Fig. S4. MALDI-TOF MS analysis of compound **18** incubated at 25, 50 and 80 °C.

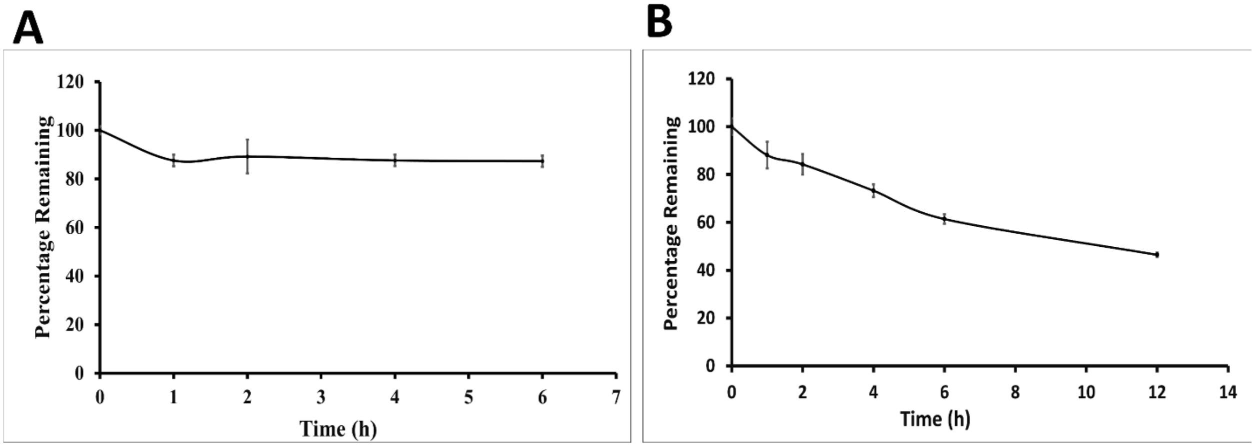

Fig. S5. Stability of **18** in A) Simulated gastric fluid (SGF) with pepsin and, B) simulated intestinal fluid (SIF). Compound **18** was incubated in SGF and SIF, and aliquots were withdrawn at selected times and analyzed by HPLC. Zero time point peak area was taken as 100%, and other time point data are represented as percentage of zero time point area. For details see the experimental section.

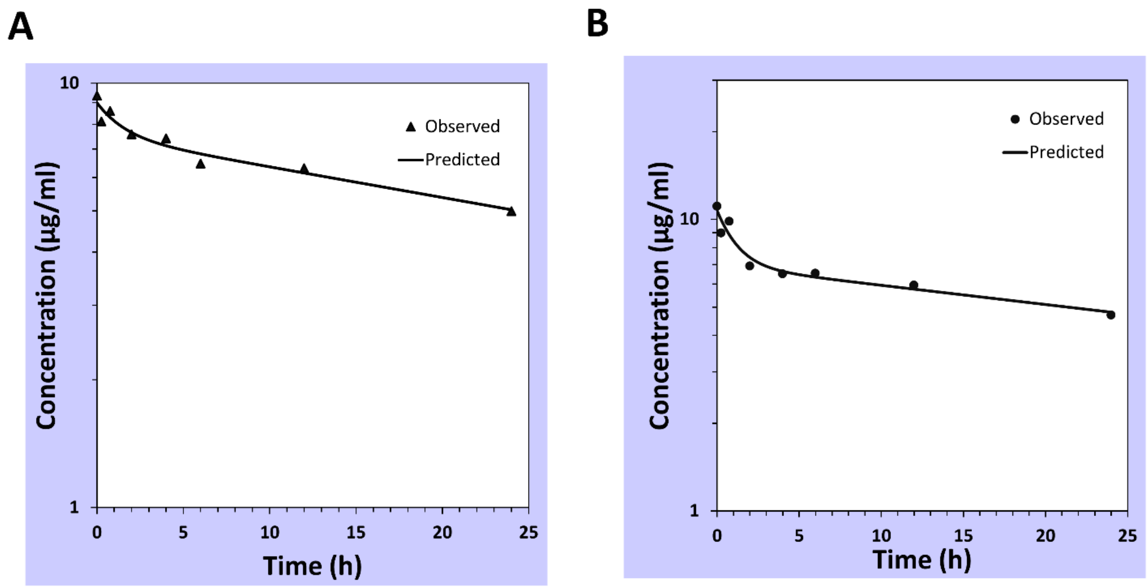

Fig. S6. Pharmacokinetic studies of **18** in Foxn1 nude mice. PK studies were performed at two doses. Compound **18** in PBS was injected into mice via tail vein, and blood samples were collected at different intervals. Samples were lyophilized after precipitation with cold methanol and analyzed by HPLC. Leuprolide was used as an internal standard. AUC was calculated and concentration of compound **18** was calculated from a standard curve. Plot of log of concentration of **18** vs. time at doses of A) 4 mg/kg and B) 6 mg/kg. PKsolver was used to curve-fit the data points according to a standard two-compartment model. The solid curve is the data predicted from the model, points are for observed data.

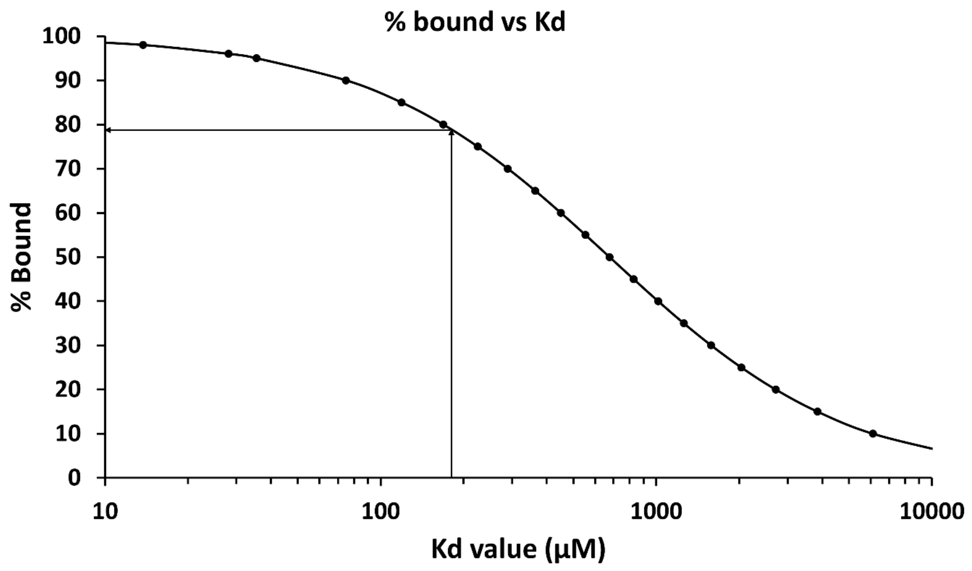

Fig. S7. Calculation of percentage of compound **18** bound to human serum albumin (HSA) based on the equation described by Rich et al., *Analytical Biochemistry* **296**, 197-207 (2001). The K_d value for compound **18** binding to HSA used for this calculation was obtained from surface plasmon resonance (SPR) analysis.

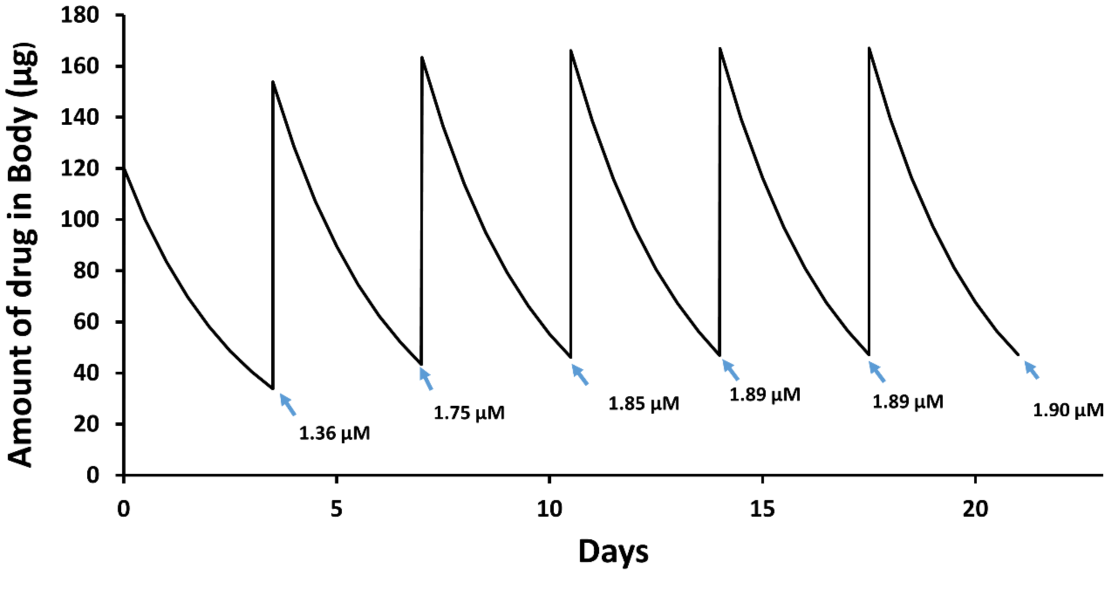

Fig. S8. Simulation of the amount of compound **18** in mice based on pharmacokinetic data for IV dosing. To maintain the concentration of compound **18** above the IC_{50} value (from the antiproliferative activity) twice-a-week dosing was chosen in the in vivo cancer model in mice.

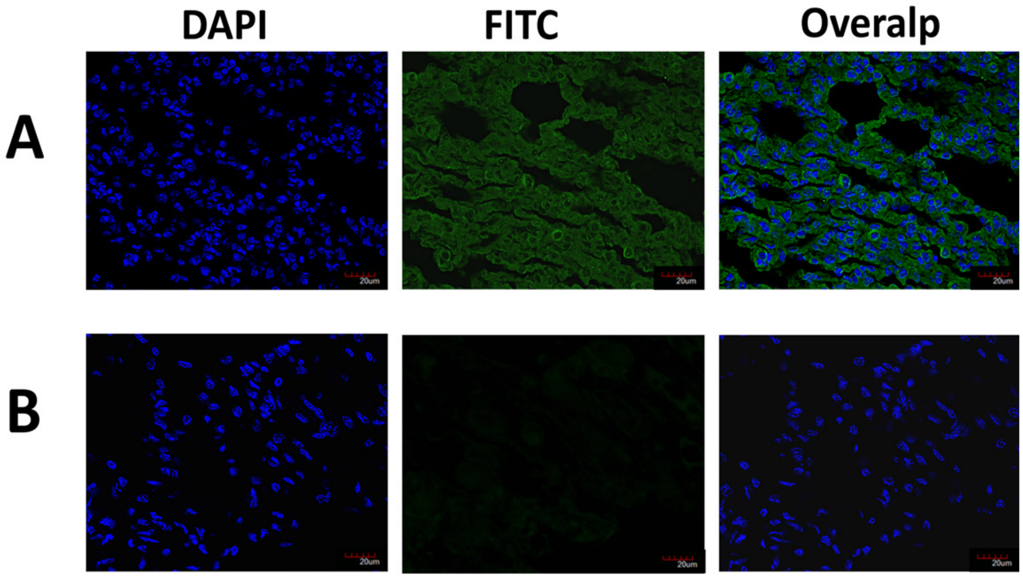

Fig. S9. Evaluation of expression of HER2 in A) lung tissue of mice that developed cancer using A549 cells. B) Lung tissue of mice that were not injected with A549 (control). HER2 expression was evaluated using an HER2 affibody with fluorescent label FITC. Note the green fluorescence in mice that were injected with A549 cells indicating HER2 overexpression. In normal mouse lung tissue HER2 overexpression was not seen (B). Magnification 40 ×, scale bar 20 μm.

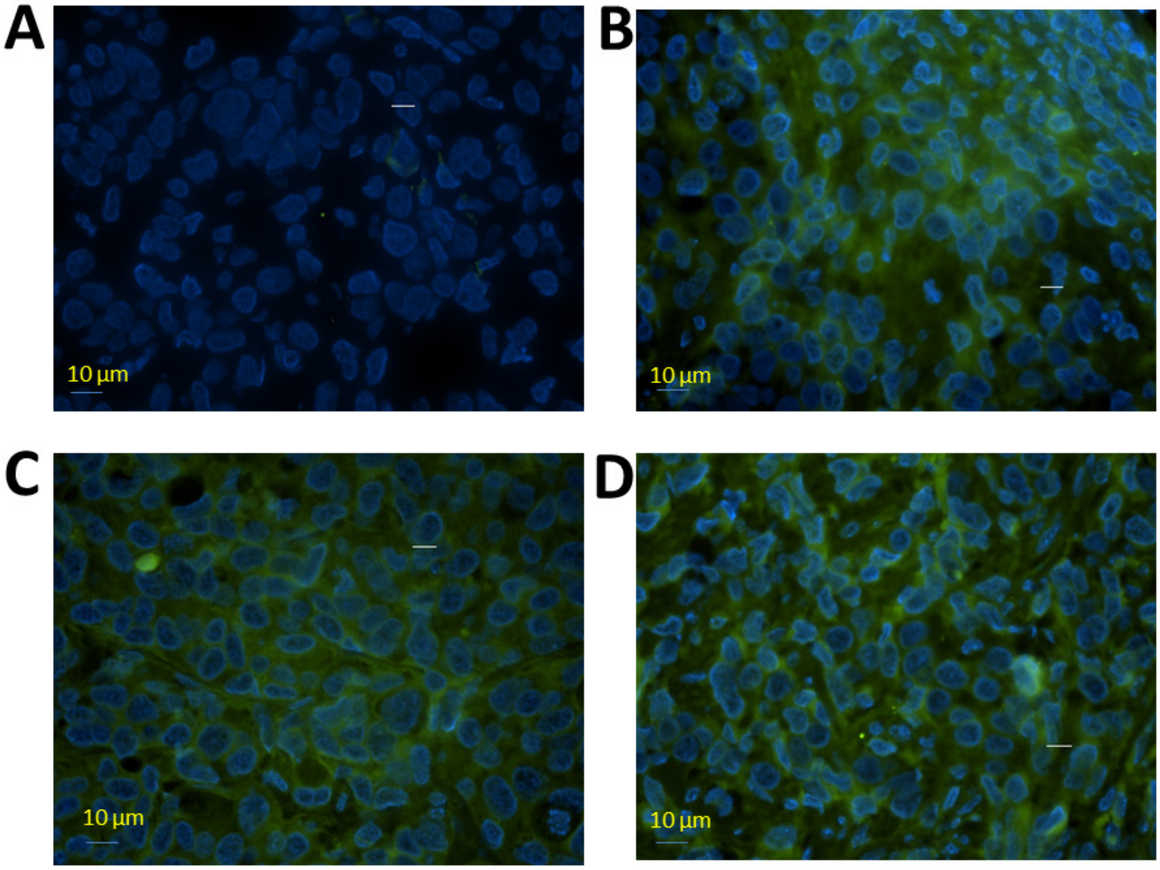

Fig. S10. Evaluation of HER2 expression in HER2-positive breast cancer tissue samples from xenograft model mice. Green fluorescence is indicative of HER2 expression in tumor sections evaluated by fluorescently labeled antibody to HER2 extracellular domain. A) Tissue section without any antibody treatment and labeled with the nuclear stain DAPI; B) in tumor tissue of control mice without treatment; C) with compound **18** treatment at 4 mg/kg via intravenous (IV) injection; D) with lapatinib treatment at 10 mg/kg intraperitoneal (IP) injection. There was no noted change in HER2 expression upon compound **18** treatment as compared to without treatment, indicating that HER2 expression is not altered by compound **18** treatment in the xenograft model tumor tissue. Scale bar: 10 µm.
